# Molecular Logic of Spinocerebellar Tract Neuron Diversity and Connectivity

**DOI:** 10.1016/j.celrep.2019.04.113

**Published:** 2019-05-28

**Authors:** Myungin Baek, Vilas Menon, Thomas M. Jessell, Adam W. Hantman, Jeremy S. Dasen

**Affiliations:** 1 Neuroscience Institute, Department of Neuroscience and Physiology, NYU School of Medicine, New York, NY 10016, USA; 2 Department of Brain and Cognitive Sciences, DGIST, Daegu 42988, Republic of Korea; 3 Janelia Research Campus, Howard Hughes Medical Institute, Ashburn, VA 20147, USA; 4 Department of Neurology, Columbia University, New York, NY 10032, USA; 5 Departments of Neuroscience and Biochemistry and Molecular Biophysics, Columbia University Irving Medical Center, New York, NY 10032, USA; 6 Lead Contact

## Abstract

Coordinated motor behaviors depend on feedback communication between peripheral sensory systems and central circuits in the brain and spinal cord. Relay of muscle- and tendon-derived sensory information to the CNS is facilitated by functionally and anatomically diverse groups of spinocerebellar tract neurons (SCTNs), but the molecular logic by which SCTN diversity and connectivity is achieved is poorly understood. We used single-cell RNA sequencing and genetic manipulations to define the mechanisms governing the molecular profile and organization of SCTN subtypes. We found that SCTNs relaying proprioceptive sensory information from limb and axial muscles are generated through segmentally restricted actions of specific *Hox* genes. Loss of *Hox* function disrupts SCTN-subtype-specific transcriptional programs, leading to defects in the connections between proprioceptive sensory neurons, SCTNs, and the cerebellum. These results indicate that Hox-dependent genetic programs play essential roles in the assembly of neural circuits necessary for communication between the brain and spinal cord.

## In Brief

Baek et al. show that Hox-transcription factor-dependent programs govern the specification and connectivity of spinal interneurons that relay muscle-derived sensory information to the cerebellum. These findings shed light on the development of neural circuits required for proprioception—the perception of body position.

## Graphical Abstract

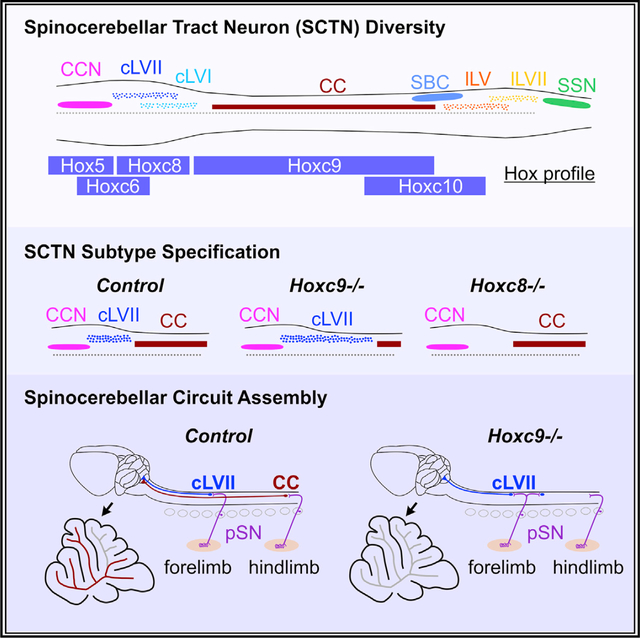

## INTRODUCTION

Relay of muscle-derived sensory information from the periphery to the CNS is essential for coordinating motor output during behavior and plays essential roles during motor learning and adaptation (Bosco and Poppele, 2001; Tuthill and Azim, 2018). The role of proprioception in motor control has been investigated in animal studies where sensory neurons have been genetically or surgically ablated, as well as in sensory neuropathies that disrupt proprioceptive feedback (Dietz, 2002). While basic motor functions such as walking and reaching are retained, loss of proprioception causes severe defects in limb coordination. In humans with sensory deficits, the ability to move the arm is maintained but characterized by the inability to predict and correct errors (Ghez et al., 1995; Gordon et al., 1995). Ablation of hindlimb proprioceptive input leads to a loss of inter-joint limb coordination, as well as defects in the ability of animals to adapt locomotor behaviors when confronted with uneven terrains (Abelew et al., 2000; Akay et al., 2014; Windhorst, 2007).

Muscle- and joint-derived sensory information is relayed to the CNS through specialized classes of proprioceptive sensory neurons (pSNs) that connect peripherally with muscle spindles and Golgi tendon organs (Chen et al., 2003). Centrally, pSNs establish connections with diverse arrays of neuronal subtypes, including spinal motor neurons (MNs), local circuit interneurons, and ascending projection neurons. Ascending pathways relay information related to muscle contractile status to higher brain centers, including the cerebellum. Proprioceptive sensory streams are transmitted to the cerebellum through neurons that project along the spinocerebellar and cuneocerebellar tracts (Bosco and Poppele, 2001; Popova et al., 1995). Spinal projections originating from spinocerebellar tract neurons (SCTNs) terminate as mossy fibers and constitute a major source of input to cerebellar granule cells.

Anatomical tracing studies in mammals indicate that SCTNs comprise up to a dozen distinct subtypes that are located at discrete positions along the rostrocaudal axis of the spinal cord (Arsénio Nunes and Sotelo, 1985; Matsushita and Gao, 1997; Matsushita et al., 1979; Sengul et al., 2015). Electrophysiological studies, predominantly in cats and rats, have shown that each SCTN type is targeted by pSNs that innervate specific muscle groups. For example, neurons within Clarke’s column (CC) relay proprioceptive information from hindlimb muscles, the central cervical nucleus from the neck, and Stilling’s sacral nucleus from the tail (Edgley and Grant, 1991; Kuno et al., 1973; Popova et al., 1995). While specific SCTN populations convey sensory information related to the activity of broad muscle groups, individual neurons within CC appear to receive sensory inputs from multiple, and often functionally antagonistic, limb muscle types (Knox et al., 1977; Osborn and Poppele, 1988). The information relayed from pSNs to CC may provide more global information about limb parameters, such as direction of limb movement and orientation, as opposed to muscle- specific features (Popova et al., 1995). In addition to input from pSNs, neurons within CCs receive direct excitatory and indirect inhibitory input from corticospinal neurons (Hantman and Jessell, 2010). The coincidence of cortical- and muscle-derived inputs suggests that SCTNs function as local hubs that integrate and process sensory and motor information.

Despite progress in elucidating the anatomical organization and physiological features of SCTNs, the molecular basis for their subtype diversification and connectivity is largely unknown. In principle, SCTN diversification could employ the same developmental mechanisms that have been defined for other neuronal classes, such as spinal MNs. All spinal MNs arise from a single progenitor domain but give rise to dozens of topographically organized muscle-specific subtypes (Philippidou and Dasen, 2013). This diversity is established through the activities of Hox transcription factors along the rostrocaudal axis. *Hox* genes are expressed by multiple neuronal populations within the hindbrain and spinal cord, suggesting a broader role in neuronal specification. Although recent studies have implicated *Hox* function during the differentiation of interneurons in the ventral spinal cord (Hayashi et al., 2018; Sweeney et al., 2018), the identity of their downstream target effectors and potential roles in sensory-motor circuit assembly have not been investigated.

We used single-cell RNA sequencing to define the molecular signatures of SCTNs generated at cervical and thoracic levels of the spinal cord. We show that the specification of SCTNs relies on segmental-level specific activities of Hox transcription factors, and loss of *Hox* gene function transforms the molecular profiles and connectivity patterns of SCTN subtypes. These results indicate that the specification of SCTNs relies on the same developmental programs used to generate spinal MN subtypes, suggesting a common transcriptional strategy drives cell-type diversification across multiple neuronal classes.

## RESULTS

### Organization and Input Specificity of SCTNs

**Figure 1.**
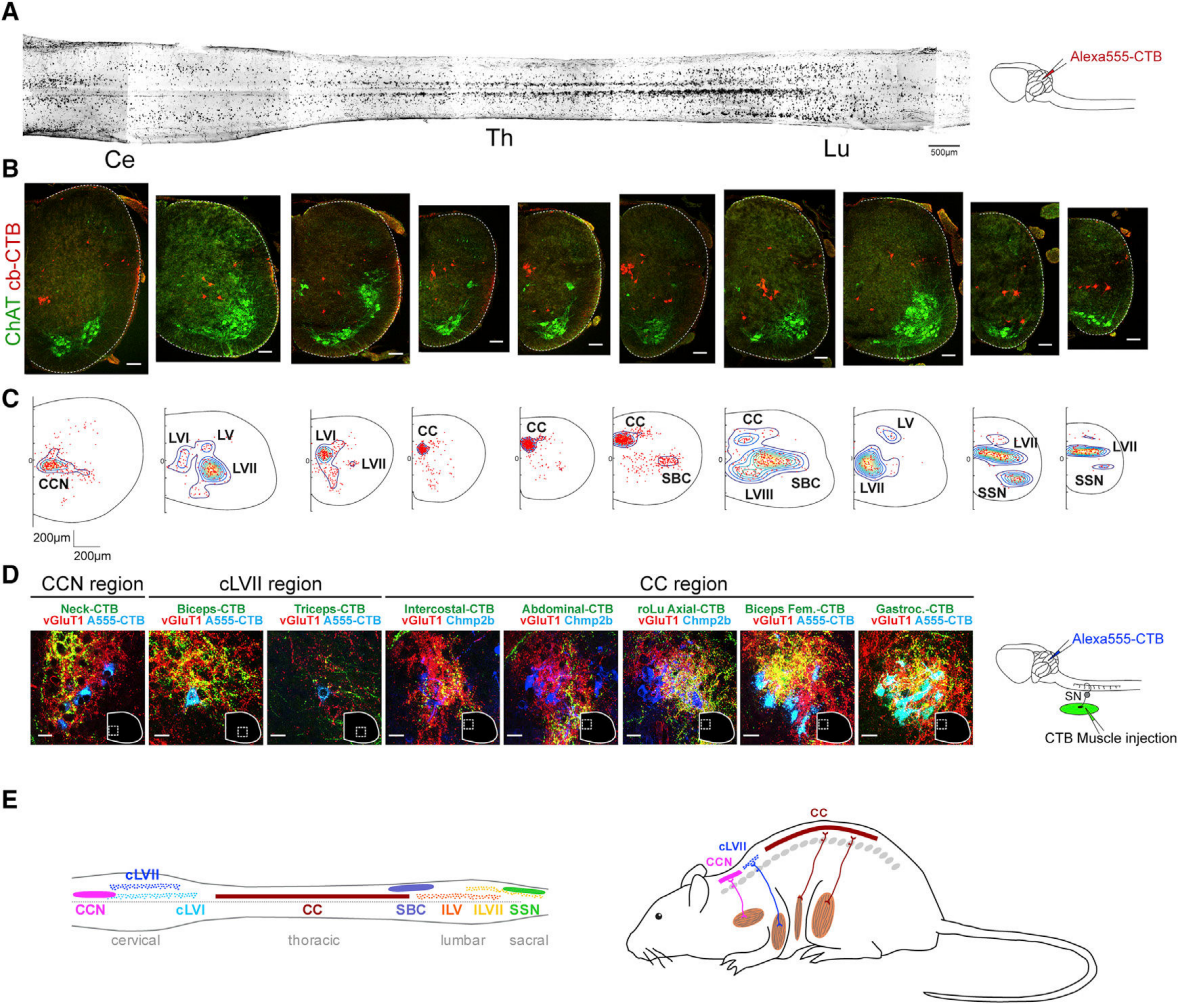
Distribution and Muscle-Specific Inputs of SCTNs (A) Whole mount of Alexa555-CTB labeled SCTNs in P6 mouse spinal cord. Injection schematic is shown on the right. Ce, cervical; Th, thoracic; Lu, lumbar. (B) CTB-labeled SCTNs in spinal cord sections. Shown are the matched regions to the whole-mount spinal cord. Last two sections are from sacral regions. Choline acetyl transferase (ChAT) staining indicates MN position. Scale bars, 100 μm. (C) Density plots of labeled SCTNs. Contour plots were generated from n = 6 spinal cords. Number of cells in each section, from left to right (rostral cervical to sacral), is 299, 165, 251, 241, 376, 662, 266, 78, 161, and 92. Distance, μm. (D) Sensory inputs to SCTNs traced by CTB injection into indicated target muscle. Shown are the magnified images of regions demarked by white dashed lines. VGluT1 labels pSN terminals, A555-CTB labels traced SCTNs, and Chmp2b marks CC neurons (found in Allen brain atlas). Injection schematic is shown on the right. Scale bars, 25 μm. (E) Summary of SCTN organization in mouse. Images in (A) and (D) are tiled composites made in Zen software. See also Figure S1.

To dissect the molecular profiles of SCTN subtypes, we first used retrograde tracing from the cerebellum to map the position of SCTNs along the rostrocaudal axis of the spinal cord. We injected Alexa555 conjugated cholera toxin B (CTB) into the cerebellum of P4 mice and allowed SCTNs to be labeled for 2 days. Whole-mount staining of the spinal cord labeled specific subsets of neurons along the rostrocaudal axis (Figure 1A). Prominent columns of neurons were found near the midline of rostral cervical, thoracic, and rostral lumbar levels and more laterally positioned columns at caudal lumbar and sacral levels. More scattered SCTN populations were found throughout the entire length of the spinal cord. We mapped the distribution of SCTNs within specific spinal segments and generated contour maps of SCTN densities at cervical, thoracic, lumbar, and sacral levels (Figures 1B and 1C). Consistent with previous studies, four prominent clusters of SCTNs were labeled, including the central cervical nucleus (CCN) at rostral cervical levels, CC neurons extending from thoracic to rostral lumbar levels, spinal border cells (SBCs) at lumbar levels, and Stilling’s nucleus (SSN) at sacral levels (Edgley and Grant, 1991; Matsushita et al., 1979; Sengul et al., 2015). We also identified SCTNs showing more distributed patterns at cervical levels in Rexed lamina (L)V, LVI, and LVII and at lumbar levels in LV, LVII, and LVIII (Figure 1C). Collectively, these tracing data identify 10 major groups of SCTNs in early postnatal mice (Figures 1E; Figure S1).

SCTNs are essential for relaying proprioceptive sensory information from muscle to cerebellum, but the muscle-specific inputs that SCTNs receive are largely unmapped in mouse. We examined the source of inputs from pSNs to SCTNs by injection of CTB into specific muscles while in parallel labeling SCTNs with either cerebellar retrograde tracing or using SCTN-restricted molecular markers. Selectivity of proprioceptive inputs was further delineated by localization with VGluT1, which labels the presynaptic boutons of pSNs (Betley et al., 2009; Shrestha et al., 2012). This analysis revealed that SCTNs receive input from discrete muscle types and are consistent with studies in rat and cat (Edgley and Grant, 1991; Mann, 1973; Popova et al., 1995; Shrestha et al., 2012). Rostral cervical CCN neurons receive inputs from pSNs innervating neck muscles, and caudal cervical LVII SCTNs receive input from forelimb muscle, while thoracic and upper lumbar CC neurons receive input from hindlimb and ventral hypaxial muscles (Figure 1B). Inputs to SBC neurons were not labeled through any of the muscle injections we attempted and did not contain VGluT1+ presynaptic boutons, as previously reported (data not shown) (Shrestha et al., 2012). These results indicate that specific populations of SCTNs can be delineated by their rostrocaudal position, settling location, and the source of their inputs from specific muscle groups.

### Molecular Profiling of SCTNs at Cervical and Thoracic Levels

**Figure 2.**
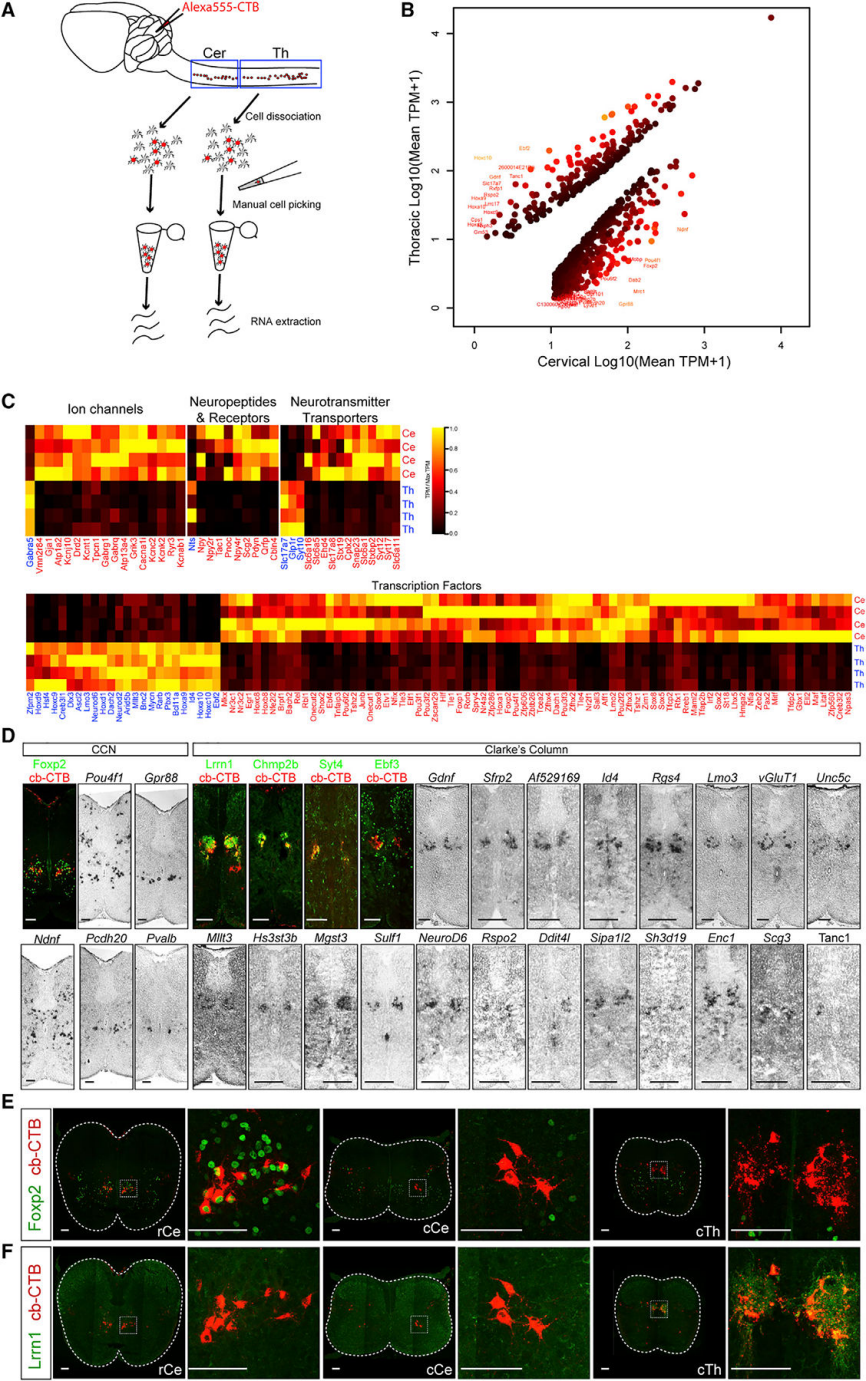
Identification and Characterization of CCN and CC Molecular Markers (A) Strategy for isolating cervical and thoracic SCTNs for RNA-seq. Isolations were performed in quadruplicate at cervical (185, 182, 179, 178 SCTNs) and thoracic (310, 305, 473, 341 SCTNs) levels. (B) Mean expression of differentially expressed genes in cervical (x axis) and thoracic (y axis) bulk RNA-seq samples. Genes with differential expression between cervical and thoracic samples with false discovery rate (FDR) < 0.001 (using edgeR), fold-change > 2, and mean transcripts per million (TPM) > 10 in either cervical or thoracic samples are shown as dots, colored by FDR value. Genes with fold-change greater than 30 are shown with text labels. (C) Heatmaps showing expression of differentially expressed genes (cervical versus thoracic, FDR < 0.001, fold-change > 2) belonging to major annotated categories. Heatmap colors represent scaled TPM values for each replicate bulk sample. (D) Validation of sequencing data by *in situ* hybridization and immunostaining. For identifying SCTNs by immunostaining, Alexa555-CTB labeled spinal cord sections were used. (E and F) Expression of Foxp2 (E) and Lrrn1 (F) in retrogradely labeled SCTNs at rostral cervical (rCe), caudal cervical (cCe), and caudal thoracic (cTh) segments. Low-magnification images in (E) and (F) are composites of tiled images generated in Zen and are matted on a black background. Images to right of these panels show higher magnification of boxed area. Scale bars in (D), (E), and (F), 100 mm. See also Figure S2.

To determine whether SCTN subtypes can be distinguished by differences in molecular profiles, we performed RNA sequencing (RNA-seq) on retrogradely labeled and individually isolated SCTNs from cervical and thoracic levels (Figure 2A). To obtain high sequencing depth, we first performed RNA-seq on pools of labeled SCTNs. We collected four pools, each containing ~200 cervical SCTNs, and four pools of ~350 thoracic SCTNs. We identified 1,768 genes that were enriched in cervical SCTNs and 495 genes enriched in thoracic SCTNs (>2-fold change; Benjamini-Hochberg [BH]-adjusted p < 0.05) (Figure 2B). Differentially expressed genes included effector molecules with implications for neural function including ion channels, neuropeptide receptors, and neurotransmitter transporters (Figure 2C). For example, selective expression of neuropeptides and associated proteins was found in cervical SCTNs (e.g., *NPY*, *Tac1*, *Pnoc*, *pdyn*, *qrfp*, and *scg2*) and thoracic SCTNs (*NTS*), suggesting that SCTN subtypes differentially release more than one neuromodulator. This dataset will be useful for testing hypotheses about anatomical and physiological differences between cervical and thoracic SCTN populations.

We further characterized genes differentially expressed between cervical and thoracic SCTNs by performing mRNA *in situ* hybridization and immunohistochemical analyses (Figure 2D). We focused on transcription factors, cell adhesion molecules, and genes implicated in neuronal function, as these classes of genes are often selectively expressed by neuronal subtypes. Most of the cervical enriched genes we identified were expressed in a cluster of neurons located in rostral cervical segments, near the position occupied by CCN neurons. Putative CCN-restricted genes included *Foxp2*, *Pou4f1*, *Gpr88*, *Ndnf*, and *Pcdh20* (Figures 2D and S2A). We confirmed selective expression of *Foxp2* in CCN neurons by performing cerebellar retrograde tracing of SCTNs in conjunction with Foxp2 antibody staining. This analysis revealed Foxp2 is expressed by labeled SCTNs at rostral cervical levels but not in caudal cervical or thoracic SCTNs (Figures 2D and 2E). We also identified a number of genes selective for thoracic CC neurons, including the previously characterized *Gdnf* and *VGlut1* genes (Hantman and Jessell, 2010). We confirmed SCTN-restricted expression of additional genes, including *Lrrn1*, *Chmp2b*, *Syt4*, and *Ebf3*, by performing *in situ* hybridization or immunohistochemistry in conjunction with cerebellar CTB tracing (Figures 2D and S2B). These genes were expressed by clusters of thoracic neurons, but not in cervical SCTNs, indicating they are selective markers for CC neurons (Figure 2F; data not shown).

### Single-Cell Molecular Profiling of SCTNs

**Figure 3.**
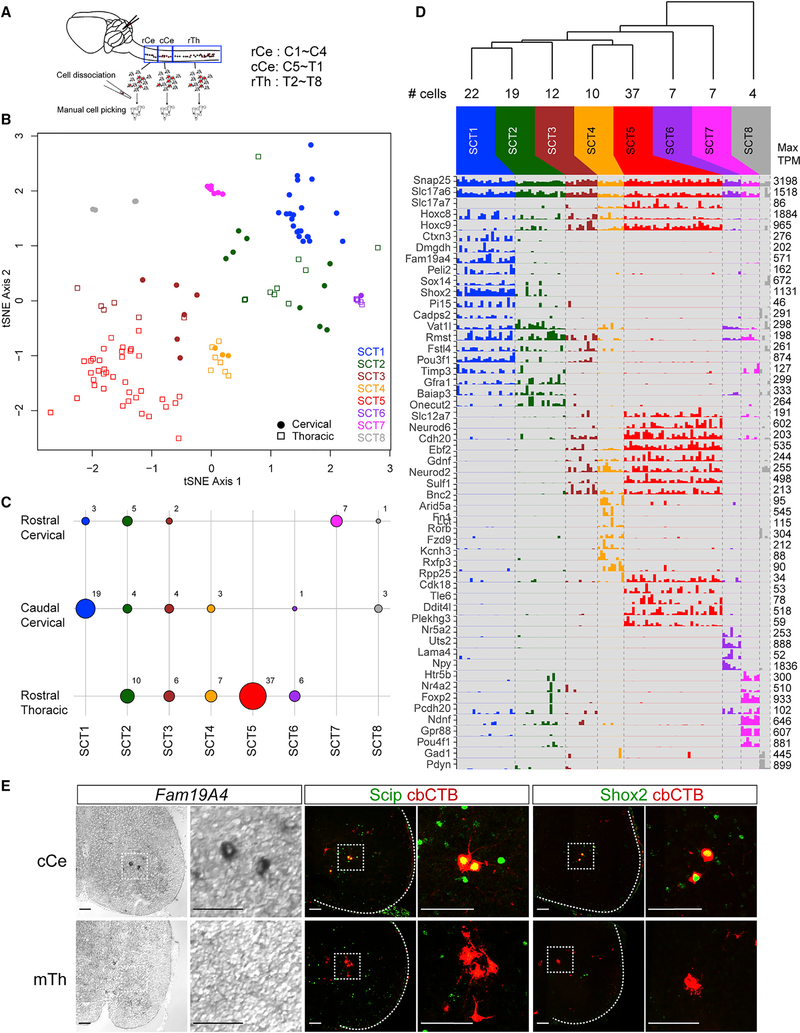
Characterization of SCTN Subtypes by scRNA-seq (A) scRNA-seq workflow. Alexa555-CTB labeled SCTNs were isolated from rostral cervical (rCe), caudal cervical (cCe), and rostral thoracic (rTh) spinal cord at P7. (B) Visualization of putative cell clusters in a t-Distributed Stochastic Neighbor Embedding (t-SNE) plot. Cells were clustered as described in the methods (not in t-SNE space), and cluster identities SCT1 through SCT8 are color-coded in the plot. Shapes represent the dissection from which cells were obtained. (C) Dot plot showing the number of cells in each cluster deriving from each segmental dissection. The size of each circle indicated the number of cells in a given cluster from a specific dissection, and the corresponding numbers are indicated to the right of the circles. (D) Barplot showing the expression (TPM) values for selected pan-class genes and genes with differential expression across clusters. The hierarchical dendrogram at the top was generated using complete linkage, with the distance metric defined as the Euclidean distance between mean log10(TPM+1) values for each cluster. For each gene, the maximum TPM value is indicated by the number to the right of each row in the bar plot. (E) Expression of *Fam19A4*, Scip, and Shox2 in cCe SCTNs. For Scip and Shox2 analyses, SCTNs were labeled by cerebellar-CTB (cbCTB) retrograde tracing. Images in (E) are tiled images generated in Zen and are matted on black background. Scale bars, 100 μm. See also Figure S3.

To further examine the diversification of SCTNs using genomewide assays and to identify smaller subgroups of SCTNs, we performed single-cell RNA-seq on neurons isolated from rostral cervical, caudal cervical, and rostral thoracic levels. We manually isolated ~100 retrogradely labeled SCTNs from each level and performed single cell RNA sequencing (scRNA-seq). Unsupervised clustering of scRNA-seq data identified eight clusters of neurons (SCT1–8) (Figures 3A and 3B; Figures S3A and S3B). Two clusters, SCT7 and SCT5, were unique to rostral cervical and rostral thoracic segments and expressed genes indicative of CCN and CC fates, respectively, based on the number and identity of genes that overlapped with our bulk sequencing analyses (Figure S3C). For example, SCT7 expresses *Foxp2* (a CCN marker), while SCT5 expresses *Gdnf* (CC marker). Two clusters, SCT2 and SCT3, were present in each of the three segmental levels we analyzed (Figure 3B), possibly representing Hox-independent populations. Four clusters (SCT1, 4, 6, and 8) were present at two levels, with higher representation within a single region. These results potentially identify additional SCTN populations that were likely masked by over-representation of CCN- and CC-restricted genes in our bulk sequence analysis.

To determine whether any of our single-cell clusters identify additional SCTN types, we chose genes within cluster SCT1 for further analysis. SCT1 neurons derive from caudal cervical segments, possibly representing the LVII SCTN subtype. SCT1 neurons are characterized by elevated expression of *Fam19A4*, *Shox2*, and *Scip* (*Pou3f1*) (Figure 3D). We found that the *Fam19A4* gene was selectively expressed in caudal cervical segments and marked a small group of spinal neurons (Figure 3E). We confirmed expression of *Fam19A4* in cervical LVII SCTNs by performing *in situ* hybridization on spinal cord sections in which SCTNs were labeled through cerebellar retrograde tracing (Figure S3D). Using this approach, we also identified the transcription factors *Shox2* and *Scip* as a selective markers for cervical LVII SCTNs. Although both proteins are expressed throughout the rostrocaudal axis of the spinal cord, we found that Shox2 and Scip were selectively expressed by cerebellar-projecting SCTNs at caudal cervical levels (Figure 3E). Collectively, our bulk and single-cell RNA-seq analyses demonstrate that three SCTN subtypes (CCN, cLVII, and CC) can be molecularly distinguished by differential gene expression.

### Hox Protein Expression Defines SCTN Subtypes

**Figure 4.**
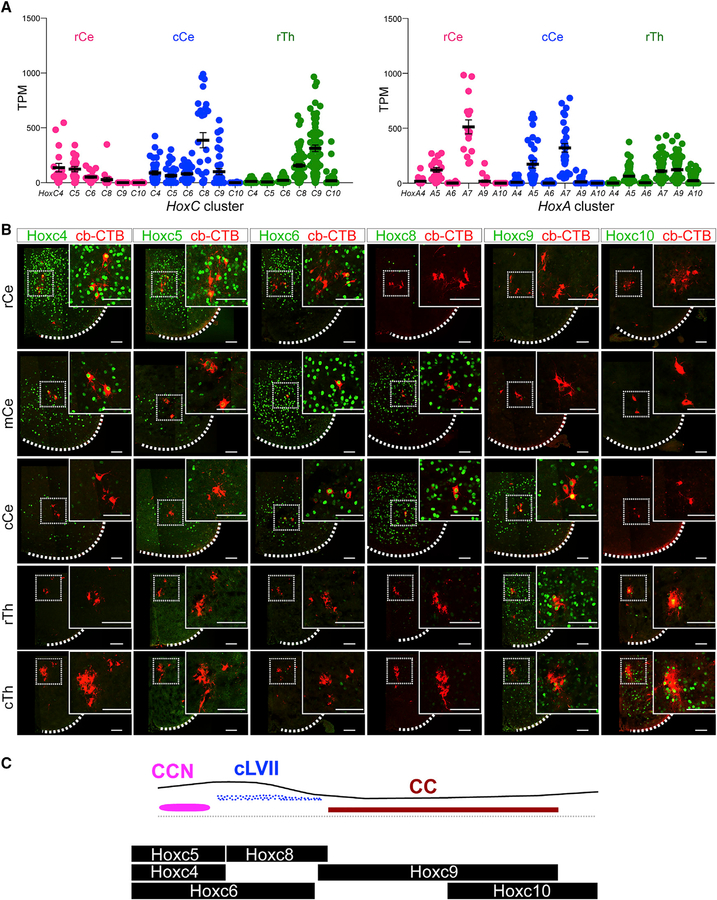
Hox Expression Patterns within SCTN Subtypes (A) Plots of scRNA-seq data showing *HoxC* and *HoxA* cluster gene expression levels (TPM) in each segmental region. Only *Hox4-Hox10* paralogs are shown, and gene names are abbreviated (e.g., C4 = *Hoxc4*). rCe, 18 cells; cCe, 34 cells; rTh 66 cells. Solid lines indicate mean TPM; error bars indicate ± SEM. (B) Hox protein expression in SCTN subtypes at cervical and thoracic levels. SCTNs were labeled by injection of Alexa555-CTB into the cerebellum at P1 and analyzed using indicated Hox antibodies at P2. Images are tiled composites generated in Zen and are matted on a black background. Scale bars, 100 μm. (C) Summary of *HoxC* gene expression in cervical and thoracic SCTNs. See also Figure S4.

What are the mechanisms that determine the diversity and molecular signatures of SCTN subtypes? Because a major difference between SCTNs is their segmental organization, we examined differences in *Hox* gene expression, known determinants of rostrocaudal patterning in the CNS (Philippidou and Dasen, 2013). In vertebrates, *Hox* genes are organized in four chromosomal clusters, and the position of individual genes within a cluster determines where it is expressed along the rostrocaudal axis. In general, *Hox* genes located at the 3^′^ end of a cluster are expressed rostrally, while those at the 5^′^ end are expressed caudally. Analysis of our scRNA-seq dataset revealed that cervical and thoracic SCTNs follow this co-linear *Hox* pattern. Rostral cervical SCTNs expressed elevated levels of *Hox4-Hox5* gene paralogs (e.g., *Hoxc4*, *Hoxc5*, and *Hoxa5*), and caudal cervical SCTNs expressed *Hox6-Hox8* paralogs (*Hoxc6* and *Hoxc8*), while rostral thoracic SCTNs express *Hox9* genes (*Hoxc9* and *Hoxa9*) (Figure 4A). In addition, certain *Hox* genes were expressed in multiple segments, suggesting specific combinations of Hox proteins contribute to SCTN specification. For example, *Hoxc8* is detected in both caudal cervical and rostral thoracic SCTNs, while *Hoxc6* is expressed by both rostral and caudal cervical SCTNs (Figure 4A; Figure S4A).

We further examined Hox protein expression by performing immunohistochemical analyses in which SCTNs were labeled by cerebellar retrograde tracing at P1. This analysis revealed that cervical CCN neurons express Hoxc4, Hoxc5, and low levels of Hoxc6 but lacked Hoxc8 and Hoxc9 expression (Figure 4B; Figure S4B). Caudal cervical SCTNs express Hoxc6 and Hoxc8, with subsets expressing Hoxc9. Thoracic CC neurons express Hox9 paralogs (Hoxa9, Hoxc9, and Hoxd9) and Hox10 paralogs (Hoxa10 and Hoxc10) (Figure 4B; Figures S4B and S4C). Collectively, these observations indicate that specific SCTNs populations can be identified by differential expression of Hox proteins and suggest specific “Hox codes” determine SCTN subtype identity (Figure 4C).

### Hox Genes Are Essential for Specifying SCTN Subtype Identity

**Figure 5.**
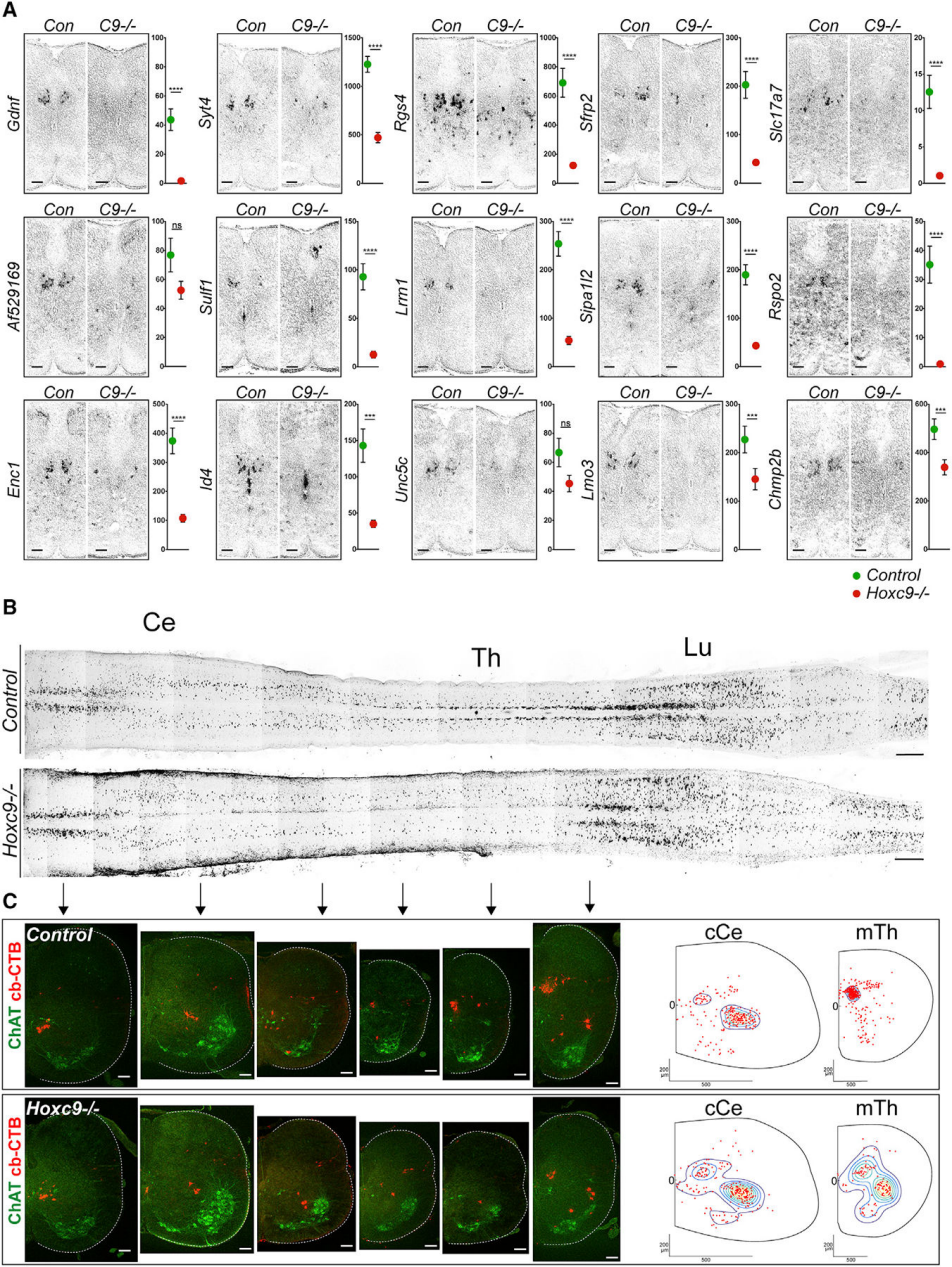
Hoxc9 Is Required for CC Neuron Development (A) *In situ* hybridization of marker gene expression in control and *Hoxc9* mutants at P6. Scale bars, 100 μm. Graphs on right show scRNA-seq data (TPM values) for each gene at thoracic levels in control and *Hoxc9* mutants (mean TPM ± SEM, ***p < 0.001, ****p < 0.0001). (B) Whole-mount images of Alexa555-CTB-labeled SCTNs in control and *Hoxc9* mutants at P6. Images in (A) and (B) are tiled composites generated in Zen and are matted on a black background. Scale bars, 500 μm. (C) Sections of Alexa555-CTB-labeled SCTNs at caudal cervical (cCe) and mid-thoracic (mTh) levels. Contour plots are shown on the right. Control cCe, 227cells, n = 8 mice; *Hoxc9*−*/*− cCe, 236 cells, n = 11 samples; Control mTh, 340 cells, n = 8 mice; *Hoxc9*−*/*− mTh, 116 cells, n = 11 mice. Scale bars, 100 μm. See also Figure S5.

To examine a possible functional role of *Hox* genes in SCTN sub type diversification, we analyzed mice in which specific *Hox* genes are mutated. We first analyzed the effects of mutation of the *Hoxc9* gene, which is normally restricted to thoracic CC neurons. Previous studies have shown that *Hoxc9* is a key determinant of MN subtype identity in thoracic segments and is essential for the generation of preganglionic autonomic MNs and repression of more anterior *Hox* genes (Dasen et al., 2003; Jung et al., 2010). We found that in *Hoxc9* mutants expression of CC-restricted genes was markedly reduced at thoracic levels (Figure 5A; Figure S5A). Markers normally displaying highly restricted expression in CC neurons, including *Gdnf*, *Syt4*, *Lrrn1*, *Unc5c*, and *Lmo3*, were undetectable in thoracic segments of *Hoxc9* mutants (Figure 5A). Genes that are expressed by CC neurons, but also other spinal populations, such as *Rgs4* and *Id4*, were lost from CC neurons but were preserved in non-SCTN populations (likely representing interneuron populations that do not rely on specific *Hox* genes or are *Hox* independent) (Figure 5A). These observations indicate that *Hoxc9* is necessary for establishing CC-specific gene programs at thoracic levels.

The loss of CC-restricted gene expression in *Hoxc9* mutants suggests *Hox* genes are generally required for deployment of SCTN-subtype-specific programs. To further explore this idea, we examined whether additional *Hox* genes are essential during SCTN diversification. We examined the function of *Hoxc8*, which is expressed by caudal cervical LVII SCTNs and characterized by selective expression of *Fam19A4*. We found that in *Hoxc8* mutants, expression of *Fam19A4* was lost from the spinal cord (Figure S5B). Interestingly, expression of Scip and Shox2 were retained by some caudal cervical SCTNs (Figure S5C; data not shown), possibly a result of functional compensation by other *Hox* genes. These results indicate that *Hox* genes are essential for the normal specification of SCTN subtypes at cervical and thoracic levels.

The depletion of SCTN markers in *Hox* mutants could be due to the death of these populations at specific segmental levels or a fate switch to an alternate SCTN identity. To assess this at a cellular level, we performed cerebellar retrograde tracing to determine whether any SCTNs are generated in thoracic segments of *Hoxc9* mutants. We injected CTB into the cerebellum of *Hoxc9* mutants and mapped the position of labeled SCTNs. We found that in *Hoxc9* mutants the dorsomedial population of CC neurons is no longer labeled in thoracic segments, with only a small population present at rostral lumbar levels (Figures 5B and 5C). SCTNs were labeled in thoracic segments but were scattered and resided in a position similar to those of caudal cervical LVII types (Figures 5B and 5C) and were reduced in number (11 ± 1 [mean ± SEM] SCTNs in *Hoxc9* mutants [n = 11 animals] versus 42 ± 6 in controls [n = 8] from rostral to mid- thoracic segments, p < 0.001). In contrast, the pattern and number of labeled of SCTNs at caudal cervical levels was similar between control and *Hoxc9* mutants (21 ± 4 SCTNs in *Hoxc9* mutants [n = 11] versus 28 ± 3 in controls [n = 8] at caudal cervical levels, not significant [n.s.]), indicating a selective function of *Hoxc9* in thoracic SCTNs. These results indicate that in the absence of *Hoxc9*, thoracic SCTNs acquire the settling characteristics of cervical LVII SCTNs.

### CC Is Transformed to a Cervical SCTN Identity in Hoxc9 Mutants

**Figure 6.**
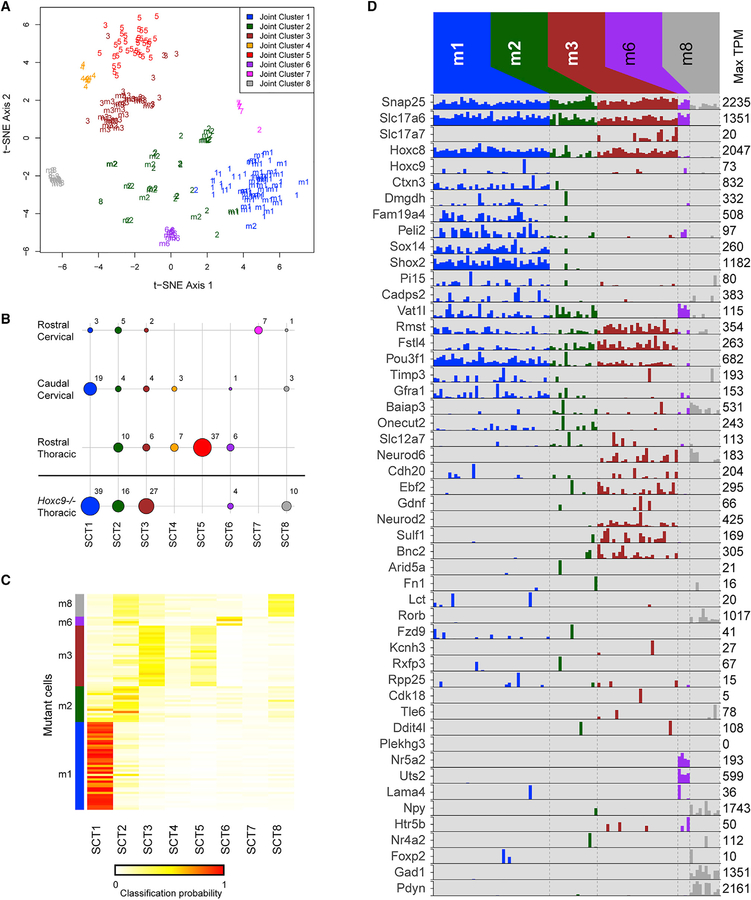
Single-Cell RNA Sequencing of Thoracic SCTNs in Hoxc9 Mutants (A) t-SNE visualization of putative joint and separate cell clusters using all cells from both control and *Hoxc9* mutants. Cells were clustered in three sets: (1) control only (as represented in Figure 3), which are labeled as 1–8 on the plot, corresponding to SCT1 through SCT8, (2) mutant-only, which are labeled as m1, m2, m3, m6, and m8 on the plot, and (3) both control and mutant cells; these joint clusters are color-coded on the plot. In general, the joint clusters agree with the independent clustering of control-only and mutant-only cells and suggest the correspondence across the two sets of cells. For example, joint cluster 1 (blue) contains cells mostly from control SCT1 (1) and mutant cluster 1 (m1), while joint cluster 3 (brown) contains cells mostly from control SCT3 (3) and mutant cluster 3 (m3). (B) As in Figure 3C, dot plot representing the number of cells in each cluster originating from each control and mutant dissection. For the mutant, each cluster was assigned to its corresponding control SCT cluster, based on the joint clustering shown in (A). The numbers for the control dissections are the same as in Figure 3C. (C) Alternative approach to assign *Hoxc9* mutant cells to control clusters. The heatmap shows the classification probabilities for each mutant cell (row) using a random forest classifier trained on the eight control cluster identities (columns). The colorbar on the left indicates the mutant cell cluster identity (m1, m2, m3, m6, and m8). The overall classification closely resembles the result from the joint clustering shown in (A); for example, cells from m1 have high classification scores for control SCT1, whereas cells from m3 tend to be most strongly assigned to SCT3. (D) As in Figure 3D, barplot showing expression (TPM) values for selected genes in the cell clusters derived from *Hoxc9* mutant SCTNs. See also Figure S6.

The acquisition of LVII neuron characteristics by thoracic SCTNs suggests a possible identity transformation in *Hoxc9* mutants. To examine a potential fate conversion at a molecular level, we assessed global changes in the transcriptomes of SCTNs in absence of *Hoxc9* function. We compared scRNA-seq profiles from rostral thoracic SCTNs isolated from control and *Hoxc9* mutants and compared these with control rostral and caudal cervical SCTN populations (Figure 6A). We found that rostral thoracic SCTNs lacking *Hoxc9* failed to form the CC cluster (SCT5) and the transcript levels of CC-restricted genes were markedly reduced (Figure 5A). The molecular profile of many thoracic SCTNs in *Hoxc9* mutants matched those of caudal LVII SCTNs (SCT1) (Figures 6B and 6C). Upregulated genes in *Hoxc9* mutants included those we identified in our scRNA-seq of control caudal cervical SCTNs, including *Hoxc8*, *Fam19A4*, *Scip* (*Pou3f1*), and *Shox2* (Figure 6D). SCT3, which is normally found at all segmental levels, was still present in thoracic SCTNs of *Hoxc9* mutants (Figure 6B), consistent with a specification program that is independent of a specific *Hox* gene or relies on more generic *Hox* activity. These results indicate that in absence of *Hoxc9*, thoracic CC neurons acquire the molecular profile of cervical SCTNs.

**Figure 7.**
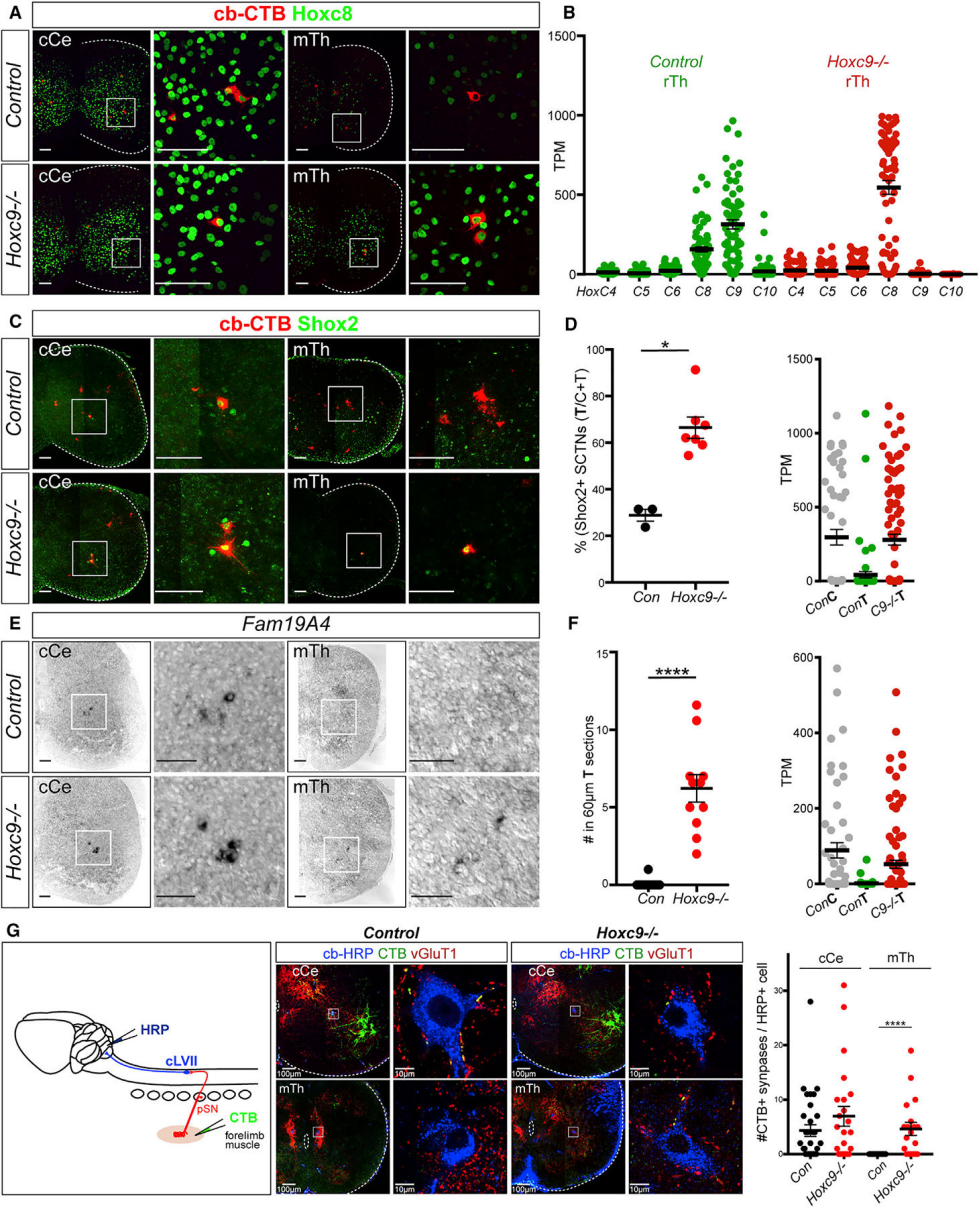
Transformation of SCTN Identity and Connectivity in Hoxc9 Mutants (A) Immunostaining of Hoxc8 in retrogradely labeled SCTNs in control and *Hoxc9* mutants in cCe and mTh segments. Thoracic SCTNs express Hoxc8 in *Hoxc9* mutants. (B) Quantification of *Hox* gene expression in single cells from control and *Hoxc9* mutant thoracic regions. Solid lines indicate mean; error bars indicate SEM. (C) Ectopic expression of Shox2 in thoracic SCTNs of *Hoxc9* mutants. (D) Quantification of Shox2^+^ SCTN number and TPM scRNA-seq values of ConC, ConT, and C9−/−T regions. Wilcoxon-Mann-Whitney test, *p = 0.0167; Con, n = 3, 91cells (Ce, 64; Th, 27)*; Hoxc9*−*/*−, n = 7, 179 cells (Ce, 59; Th, 120). For the Shox2+ cell quantification, cells were counted in regions belong to LVII group according to contour plot in Figure 1. (E) Ectopic expression of *Fam19A4* in thoracic sections of *Hoxc9* mutants. (F) Quantification of *Fam19A4*+ cells and TPM single-cell values of ConC, ConT, and C9−/−T regions. Wilcoxon-Mann-Whitney test, ****p < 0.0001; Con, n = 4 (P6, 2; E15.5, 2; 21 sections); *Hoxc9*−*/*−, n = 2 (P6, 1; E15.5, 1; 11 sections). (G) Forelimb pSNs synapse with thoracic SCTNs in *Hoxc9* mutants. Middle panels show immunostaining of HRP, CTB, and vGlut1. Right panel shows quantification of synapses between forelimb pSNs and SCTNs. For control cCe, mutant cCe, control mTh, and mutant mTh, synapses were counted in cells belonging to LVII and CC group according to contour plot in Figure 1. Wilcoxon-Mann-Whitney test, ****p < 0.0001. Images in (A), (C), and (G) are tiled composites generated in Zen and are matted on a black background. Scale bars in (A), (C), and (E): 100 mm. See also Figures S7 and S8.

To further characterize the transformation of CC neurons in *Hoxc9* mutants, we examined whether genes normally enriched in caudal cervical SCTNs are derepressed at thoracic levels. Consistent with our scRNA-seq data, as well as previous studies on *Hoxc9* function in spinal MNs, Hoxc8 protein was derepressed in thoracic SCTNs of *Hoxc9* mutants (Figures 7A and 7B). Retrograde tracing of SCTNs in *Hoxc9* mutants confirmed that labeled thoracic SCTNs ectopically express Hoxc8 (Figure 7A). In addition, expression of Hoxc10 was lost from SCTNs at thoracic levels (Figure S7A). We also analyzed expression of Scip and Shox2 proteins, two markers enriched in caudal cervical SCTNs. The number of thoracic SCTNs expressing Scip and Shox2 was markedly increased in *Hoxc9* mutants (9 ± 2 [mean ± SEM] Shox2^+^ SCTNs per section in controls [n = 3] versus 17 ± 2 in [n = 7] *Hoxc9* mutants, and 8 ± 3 Scip^+^ SCTNs in controls [n = 3] versus 17 ± in [n = 6] *Hoxc9* mutants) (Figures 7C, 7D, S7B, and S7C). In addition, *Fam19A4*, a selective marker for caudal cervical SCTNs, was ectopically expressed in *Hoxc9* mutants (Figures 7E and 7F). The transformation of CC neurons to a cervical LVII fate was also observed in rostral thoracic segments of *Nestin::Cre;Hoxc9 flox/flox* mice, indicating this identity switch is due to a neural-specific function of *Hoxc9* and not general defects in early rostrocaudal patterning (Figures S7D–S7F). Although we cannot formally rule out a selective loss of SCTN number as a contributing factor to the phenotype of *Hoxc9* mutants, our results indicate that a subset acquire both the anatomical settling position and molecular identity of caudal cervical LVII neurons.

We also asked whether loss of *Hoxc8*, which is required for acquisition of cervical LVII SCTN molecular features, leads to a similar transformation in identity. In *Hoxc8* mutants, Hoxc4 and Hoxc5 were derepressed in caudal cervical segments (Figure S5D). In addition retrograde tracing from the cerebellum indicated that labeled caudal cervical SCTNs ectopically express *Hoxc4*, suggesting a fate switch to a more rostral identity (Figure S5E). However, analysis of CCN marker expression, including *Foxp2* and *Gpr88*, failed to reveal a transformation in SCTN identity (data not shown). The absence of a complete fate transformation in *Hoxc8* mutants is likely due to presence of additional *Hox* genes in caudal cervical segments, leading to an ambiguous Hox code.

### Transformation of SCTN Identity Disrupts Spinocerebellar Circuitry

Our results indicate that in the absence of *Hoxc9*, thoracic SCTNs are converted to a cervical LVII SCTN molecular identity. We examined whether this switch in transcriptional profile is accompanied by changes in the connectivity between SCTNs, pSNs, and the cerebellum. We first assessed whether the loss of CC identity in *Hoxc9* mutants affects innervation of the cerebellum by SCTN axons. Because the number of thoracic SCTNs is markedly reduced in *Hoxc9* mutants (Figure 5B), we tested whether there is an overall loss of innervation. To label precerebellar SCTN axons, we injected an adeno-associated virus (AAV) expressing GFP under the *synaptophysin* promoter into rostral cervical and thoracic segments and examined axonal termination patterns (Figure S8A). In control animals, injections into rostral cervical segments (containing CCN neurons) labeled axons that terminate in lobules 2, 3, 4/5, and 9. Injection of viral tracer into thoracic segments exhibited denser cerebellar innervation that terminated in lobules 2, 3, 4/5, 8 and 9. In *Hoxc9* mutants, the overall density of projections from thoracic segments to the cerebellum was markedly reduced (Figures S8A and 8B). These observations indicate that loss of *Hoxc9* erodes the normal profile of connectivity between thoracic SCTNs and the cerebellum.

Caudal cervical LVII SCTNs receive input from pSNs that target forelimb muscle. If the transformation of CC neurons to a caudal cervical LVII identity switches their connectivity, they might now receive ectopic inputs from the central afferents of forelimb pSNs. We therefore examined whether ectopic thoracic LVII SCTNs receive forelimb muscle input. We injected CTB into forelimb muscles of control and *Hoxc9* mutant animals while in parallel tracing SCTNs through injection of HRP into the cerebellum. Synapses between CTB-traced proprioceptors onto HRP+ SCTNs was determined by costaining with VGluT1. The number of ectopic synapses from limb proprioceptors to thoracic SCTNs was markedly increased in *Hoxc9* mutants (5 ± 1 [mean ± SEM] CTB^+^ synapses/HRP^+^ SCTN in *Hoxc9* mutants [19 cells from n = 4 animals] versus 0 ± 0 in controls [24 cells from n = 4 animals], p < 0.0001) (Figure 7G). These results indicate that the transformed SCTNs in *Hoxc9* mutants receive presynaptic inputs appropriate for their switch in identity. Because cervical sensory afferents normally project into thoracic spinal segments (Baek et al., 2017), this switch in connectivity is likely due to alterations in the local selection of postsynaptic targets and not a consequence of broad changes in sensory central projections. Collectively, these results show that *Hox* genes are essential for the subtype diversification and connectivity of neurons within spinocerebellar circuits.

## DISCUSSION

Control of movement depends on accurate reporting of muscle and joint contractile status from pSNs to the CNS. Proprioceptive information is relayed to the cerebellum through diverse SCTN subtypes (Edgley and Grant, 1991; Matsushita et al., 1979; Sengul et al., 2015), but the molecular logic by which SCTN identity and connectivity is achieved is largely unknown. By combining single-cell molecular profiling and genetic analyses, we have identified a Hox-dependent genetic program essential for the diversification and synaptic specificity of SCTNs that relay proprioceptive sensory information from limb and axial muscle to the cerebellum. Our findings indicate that the same developmental mechanisms used to generate the diversity of spinal MNs are essential for specifying subtypes of sensory-relay interneurons. These results suggest a general mechanism through which a single large family of transcription factors establishes the diversity of multiple neuronal classes.

### Molecular and Anatomical Diversity of SCTNs

Using genome-wide interrogation of SCTN subtypes generated at cervical and thoracic levels, we identified molecular signatures that distinguish CCN, cLVII, and CC neurons, three major SCTN subtypes that relay proprioceptive information from neck, forelimb, and hindlimb muscles, respectively. Our scRNA-seq analysis identified eight clusters of neurons, each likely representing a specific SCTN subtype. We found that three of these clusters, SCT1, SCT5, and SCT7 represent cLVII, CC, and CCN subtypes and constitute the majority of SCTN populations generated at cervical and thoracic levels. The additional five clusters we identified could represent smaller subtypes of SCTNs, such as the more scattered populations normally observed at multiple segmental levels. The relatively small number of neurons represented in these clusters precludes definitive identification of their specific SCTN identity. Nevertheless, these populations could encompass SCTN lineages derived from spinal progenitors expressing the transcription factor *Atoh1* (Bermingham et al., 2001; Rose et al., 2009), which includes a population recently shown to define a distinct group of non-CC SCTNs (Yuengert et al., 2015).

### Role of Hox Genes in Determining SCTN Organization and Subtype-Specific Features

Our studies indicate that Hox transcription factors play critical roles in specifying SCTN subtype identity at cervical and thoracic levels. We found that SCTN subtypes can be defined by expression of specific Hox transcription factors. CCN neurons express *Hox5* paralogs, and cLVII neurons express *Hoxc8*, while CC neurons express *Hox9* and *Hox10* genes. Mutation in the thoracic *Hoxc9* gene leads to a loss of CC-specific molecular programs, while mutation in *Hoxc8* erodes the molecular specification of cLVII neurons. In the absence of *Hoxc9*, all molecular features of thoracic CC neuron are depleted, with only lumbar-level expression of these genes being maintained. The preservation of CC identity at lumbar levels suggests multiple *Hox* genes are involved in specifying CC features, which may include additional genes in the *Hox9* and *Hox10* paralog groups. Similarly, the regulation of rostral cervical CCN-restricted determinants likely requires the activities of multiple *Hox5* paralogs.

Recent studies suggest that molecular programs acting along the rostrocaudal axis play key roles in establishing subtype-specific features of spinal interneuron classes. Both V1 and V2a interneuron classes are generated from a single progenitor domain but give rise to dozens of molecularly distinct subtypes, which can be defined through differences in settling position, connectivity, and transcription factor gene expression (Bikoff et al., 2016; Francius et al., 2013; Hayashi et al., 2018; Sweeney et al., 2018). While studies of V1 interneurons have demonstrated an important role of *Hox* genes in patterning transcription factor expression (Sweeney et al., 2018), the identities of their subtypespecific targets and roles in circuit assembly are unclear. We found that in the absence of *Hoxc9*, expression of dozens of CC-restricted markers are markedly reduced. In both *Hoxc8* and *Hoxc9* mutants, more rostrally expressed *Hox* genes are derepressed, similar to the boundary-maintenance function of Hox proteins observed in MNs (Philippidou and Dasen, 2013). This leads to either a transformation in SCTN fate as in *Hoxc9* mutants or a disruption in normal specification programs, as seen in *Hoxc8* mutants. These findings suggest that similar to MNs, the diversification of spinal interneuron classes relies on Hox-dependent transcriptional networks to both activate and repress repertoires of subtype-specific genes.

### Establishing Synaptic Specificity in Proprioceptive Sensory Circuits

Our studies provide insights into developmental mechanisms through which proprioceptive circuits are assembled. After entering the spinal cord, pSNs establish highly specific connections to diverse classes of postsynaptic targets. The best-studied pSN connections are those established with MNs (Chen et al., 2003; Dasen, 2009). Each pSN forms a specific connection to the MN pool that targets the same or functionally related muscle, while avoiding MNs targeting antagonistic muscles. These connections are highly selective, such that a single pSN targets each of the ~50–100 MNs within the entire pool that supplies the same peripheral muscle (Mendell and Henneman, 1968).

How the striking synaptic specificity between pSNs and their central synaptic targets is achieved is poorly understood but appears to involve both genetic and activity-dependent processes (Mendelsohn et al., 2015; Pecho-Vrieseling et al., 2009). Mutations in genes involved in pSN fate determination, such as the transcription factors *Er81* or *Runx3*, lead to widespread defects in the connectivity and survival of pSNs (Arber et al., 2000; de Nooij et al., 2013; Inoue et al., 2002). Recent studies indicate that postsynaptic, target-derived cues shape the specificity between pSN and MNs (Sürmeli et al., 2011; Vrieseling and Arber, 2006). For example, transforming the identity of thoracic MNs to a limb-level fate, through deletion of the *Hoxc9*, causes limbderived pSNs to target MNs present at thoracic levels (Baek et al., 2017). These observations indicate that subtype identity of postsynaptic targets plays an instructive role in determining connectivity with pSNs.

In contrast to the selective connectivity between pSNs and MNs, connections between pSNs and SCTNs appear to be less specific. Neurons within CC receive direct and indirect proprioceptive inputs from multiple, often functionally antagonistic, limb muscle groups (Knox et al., 1977; Osborn and Poppele, 1988). Nevertheless, the specificity of inputs from pSNs to SCTNs could be restricted by the identity of the muscle source (e.g., forelimb versus hindlimb). We found that transformation of SCTNs identities leads to changes in their pre- and postsynaptic connectivity. In *Hoxc9* mutants, forelimb pSNs synapse with cLVII neurons ectopically generated in thoracic segments. These results parallel the circuit alterations between pSNs and MNs observed in *Hoxc9* mutants, where forelimb pSNs synapse with the ectopically generated thoracic lateral motor column MNs (Baek et al., 2017). It appears therefore that as pSN axons enter the spinal cord, target specificity is shaped by recognition of molecular differences in the subtypes of neurons they encounter. Although we cannot rule out changes in the cellular environment as a contributing factor to the altered pSN connections to SCTNs in *Hoxc9* mutants, similar changes in pSN connectivity are observed when *Hoxc9* is selectively removed from MNs (Baek et al., 2017), suggesting that sensory afferents actively seek out postsynaptic targets of the appropriate molecular identity. Similar Hox-dependent genetic programs within the spinal cord could shape synaptic specificity in multiple circuits, including descending motor and local cutaneous sensory pathways.

A notable feature of CC is an absence of registry between its segmental position and the location of the pSNs from which it receives direct input. Most CC neurons are located at thoracic levels, while hindlimb pSNs reside in lumbar segments. This positional mismatch could be attributed to a change in CC function during vertebrate evolution. One possibility is that SCTNs with CC-like molecular features were initially used for relaying proprioceptive information from axial muscle. In fish, reptiles, and amphibians, axial muscles play prominent roles in coordinating locomotor behaviors and likely required spinocerebellar pathways during motor control. The appearance of paired appendages might have attenuated the importance of axial proprioception, while hindlimb pSNs co-opted the existing thoracic system for limb-based locomotion. The *Hoxc9* gene appears to exert an important role in maintaining this ancestral SCTN genetic program, in part by suppressing expression of *Hox* genes associated with forelimb-level spinal neurons. The organization of SCTNs into clustered groups was likely a later mammalian innovation, as cervical and thoracic SCTNs of amphibians and reptiles do not appear to form longitudinal columns (Bangma and ten Donkelaar, 1982; Gonzalez et al., 1984). SCTN organization may have evolved in mammals to facilitate additional layers of interconnectivity, such as those with descending motor pathways or between different types of sensory afferents.

Studies in humans and animal models indicate that loss of muscle-derived sensory information does not prohibit the ability of spinal circuits to generate basic motor output but is essential for adaptive behaviors and motor learning. The relative contributions of proprioceptive input to local spinal networks versus ascending pathways in motor control are unclear. Mice that lack muscle spindles or pSNs display defects in locomotor coordination (Akay et al., 2014; Arber et al., 2000; Tourtellotte and Milbrandt, 1998), but whether this is due to alteration in pSN connections to spinal neurons, spinocerebellar circuits, or both is unknown. The identification of selective molecular features of SCTNs should provide means to ascertain the relative contributions of spinal and supraspinal proprioceptive pathways to motor control. These studies may provide insights into how sensorymotor information is integrated at the level of the spinal cord, as well as basic insights relevant to the study of spinocerebellar ataxias.

## STAR★METHODS

### CONTACT FOR REAGENT AND RESOURCE SHARING

Further information and requests for resources and reagents should be directed to and will be fulfilled by the Lead Contact, Jeremy Dasen (jeremy.dasen@nyumc.org).

### EXPERIMENTAL MODEL AND SUBJECT DETAILS

#### Mouse Genetics

Animal work was approved by the Institutional Animal Care and Use Committee of the NYU School of Medicine in accordance with NIH guidelines. Mouse lines used were: *Hoxc9 flox* (Baek et al., 2017), *Hoxc9*−*/*− (Jung et al., 2010), *Hoxc8*−*/*− (Catela et al., 2016), *Nestin::Cre* (The Jackson Laboratory, #003771), FVB (#207, Charles River Lab). Because we observed no phenotypic differences between wild-type and *Hoxc9+/*− animals, both genotypes are considered controls. Unless indicated otherwise, all comparisons between control and *Hox* mutants were made between littermates. No phenotypic differences between male and female animals are expected, but were not formally tested.

### METHODS DETAILS

#### Immunohistochemistry

For antibody staining of sections, slides were first placed in PBS for 5 minutes to remove OCT. Sections were then transferred to humidified trays and blocked for 20–30 minutes in 0.75 ml/slide of PBT (PBS with 0.1% Triton) containing 1% Bovine serum albumin (BSA). The blocking solution was replaced with primary staining solution containing antibodies diluted in PBT with 0.1% BSA. Primary antibody staining was performed overnight at 4°C. Slides were then washed three times for 5 minutes each in PBT. Fluorophore-conjugated secondary antibodies were diluted 1:1000–1:2000 in PBT and filtered through a 0.2 μm syringe filter. Secondary antibody solution was added to slides (0.75 ml/slide) and incubated at room temperature for 1 hour. Slides were washed three times in PBT, followed by a final wash in PBS. Coverslips were placed on slides using 110 μL of Vectashield (Vector Laboratories).

Antibodies against Hox proteins have been previously described (Dasen et al., 2003, 2005).

#### *In situ* hybridization

*In situ* hybridization of tissue sections was performed as previously described using DIG labeled probes (Jung et al., 2018). For *in situ* hybridization sections were first dried for 10–15 minutes at room temperature, placed in 4% PFA, and fixed for 10 minutes at room temperature. Slides were then washed three times for 3 minutes each in PBS, and then placed in Proteinase K solution (1 μg/ml) for 5 minutes at room temperature. After an additional PFA fixation and washing step, slides were treated in triethanolamine for 10 minutes, to block positive charges in tissue. Slides were then washed three times in PBS and blocked for 2–3 hours in hybridization solution (50% formamide, 5X SSC, 5X Denhardt’s solution, 0.2 mg/ml yeast RNA, 0.1 mg/ml salmon sperm DNA). Prehybridization solution was removed, and replaced with 100 μL of hybridization solution containing 100 ng of DIG-labeled antisense probe. Slides were then incubated overnight (12–16 hours) at 72°C in humidified chambers. Primer sequences used for amplification of probes are listed in Table S1.

After hybridization, slides were transferred to a container with 400 mL of 5X SSC and incubated at 72°C for 20 minutes. During this step, coverslips were removed using forceps. Slides were then washed in 400 mL of 0.2X SSC for 1 hour at 72°C. Slides were transferred to buffer B1 (0.1 M Tris pH 7.5, 150 mM NaCl) and incubated for 5 minutes at room temperature. Slides were then transferred to staining trays and blocked in 0.75 ml/slide of B1 containing 10% heat inactivated goat serum. The blocking solution was removed and replaced with antibody solution containing 1% heat inactivated goat serum and a 1:5000 dilution of anti-DIG-AP antibody (Sigma-Aldrich). Slides were then incubated overnight at 4°C in a humidified chamber. The following day, slides were washed 3 times, 5 minutes each, with 0.75 ml/slide of buffer B1. Slides were then transferred to buffer B3 (0.1 M Tris pH 9.5, 100 mM NaCl, 50 mM MgCl_2_) and incubated for 5 minutes. Slides were then developed in 0.75 ml/slide of B3 solution containing 3.5 μl/ml BCIP and 3.5 ml/ml NBT for 12–48 hours. After color development, slides were washed in ddH_2_0 and coverslipped in Glycergel (Agilent). A more detailed *in situ* hybridization protocol is available on our lab website (https://med.nyu.edu/dasenlab).

#### SCTNs labeling

SCTNs were labeled by injecting CTB (Alexa555 conjugated form, 1μg/μl in PBS, Cat# C34775, Invitrogen) throughout the cerebellum using NanojetII (Cat# 3-000-204, Drummond Scientific Company) at P4 and examined at P6–P7. Labeled SCTNs were collected manually as described (Hempel et al., 2007) with some modifications: before pronase incubation meninges were removed as much as possible and 150–300mm transverse spinal cords slices were generated using a razor blade.

#### SCTN Bulk RNA Sequencing and Analysis

Retrograde labeled spinal cord slices were incubated in ACSF (126mM NaCl, 3mM KCl, 1.25mM NaH2PO4, 20mM NaHCO3, 20mM D-Glucose, 2mM CaCl2, 2mM MgCl2 w/ pronase) for 50min. During cell collection for bulk sequencing, neuronal activity blockers were not included in ACSF. Sorted cells were transferred to tubes containing 50ml Picopure RNA extraction buffer. RNAs were extracted and spiked in ERCCs. Sequencing libraries were prepared using NuGEN SPIA library prep kit. Quadruplicates of pooled samples were used for bulk sequencing: Cervical (C1–C8;185, 182, 179, 178cells)/Thoracic (T1–T12, 310, 305, 473, 341 cells).

All bulk RNA-seq reads were aligned to the GRCm38 (mm10) reference genome, using the STAR alignment package (Dobin et al., 2013) with default parameters. Differential gene expression analysis was carried out on the raw count data using the edgeR software package (McCarthy et al., 2012). Differentially expressed genes were called at an FDR (Benjamini-Hochberg) corrected p value <0.05. Unless stated otherwise, all expression values in figures are in transcripts per million (TPM). For genes plotted in figures, the corresponding log-fold change thresholds are indicated in the figure caption.

#### Single Cell RNA Sequencing and Analysis

During cell collection for single cell RNA sequencing, neuronal activity blockers (TTX, APV, and DNQX) were included in ACSF as described (Hempel et al., 2007). Slices were incubated in ACSF (w/ pronase) for 50min. After dissociation of labeled cells, each cell was transferred to 0.2 mL PCR 8-tube strip (1402–4700, USA Scientific) containing 3 ul lysis buffer (0.2% Triton X-100 (Cat#T8787–100ML, Sigma Aldrich) in Nuclease-free water (Cat#AM9937, Ambion) with 0.1 U/ul RNase inhibitor (Cat#30281–1, Lucigen). During cell transfer, 0.1–0.2 ul ACSF cocktail was transferred to the collection tube. Each 8-tube strip of cells was flash frozen on dry ice and kept at −80°C until sequencing experiment was performed. Number of cells used in single cell sequencing: MRT (T2–T8), 125 cells; CRT (T2–T8), 78 cells; CCC (C5–T1), 53 cells; CRC (C1–C4), 23 cells. All cells were processed and prepared for sequencing in parallel. RNaseq data is available through GEO (accession in progress).

All single-cell RNA-seq reads were aligned to the GRCm38 (mm10) reference genome, using the STAR alignment package. Reads were collapsed by Unique Molecular Identifier (UMI) on a gene- and sample-wise basis using the DropSeqtools package with standard parameters. Cells with < 4,000 genes detected were removed, and all UMI values were normalized to transcripts per million (TPM) for clustering. Given the high detection of genes and UMIs, no dropout correction was implemented before clustering. Grouping of single cells into putative clusters was performed using an iterative gene-clustering based approach implemented in the hicat clustering package (Tasic et al., 2018). Briefly, high variance genes were identified (as those with variance greater than technical noise, as defined by variance in ERCC spike-in controls) and these genes were then clustered using a variant of Weighted Gene Co-Expression Network Analysis. The gene modules derived from this clustering were used as the reduced dimensions on which to cluster cells. Cells were clustered hierarchically using a Euclidean distance metric (on the reduced dimensions), and the resulting dendrogram was divided into clusters with the cutreeDynamic R function with the cutHeight parameter set to 0.99. The resulting clusters from this tree-cutting step were then evaluated for differential gene expression, and clusters with a total log10(p value) < 100 for all differentially expressed genes (p value < 0.05) were re-merged. Clusters containing fewer than 4 cells were merged with their parent clusters. Given that the clustering in both Figure 3 (wild-type only) and Figure 6 (joint wild-type and mutant) showed clusters with mixed membership of regions and conditions, no computational batch correction was performed.

#### SCTN and Sensory Terminal Labeling

SCTNs were labeled by injecting HRP (20%, 100mg HRP (Cat# 814 407, Roche) dissolved in 1% Lysophosphatidyl choline (Cat# L4129, Sigma Aldrich) into the cerebellum and muscle sensory terminals were labeled by CTB (2% CTB; Cat# C9903, Sigma-Aldrich) injection into the muscle at P4. Samples were perfused (4% PFA), saturated with sucrose (30%), and cryosectioned at 30um. Signals were examined at P6 using immunohistochemistry.

#### Spinal AAV Injections

Retrograde AAV variant (0.5μl, *AAV-SL1-synGFP*, gift from Janelia Research Campus) was injected into the spinal cord at P1 using NanojetII and examined at P6. Injected samples were perfused (4% PFA), saturated with sucrose (30%), and cryosectioned at 40um.

#### Image Acquisition

Zeiss confocal microscope (LSM700, 20X dry or 63X oil objective lenses) was used for acquiring images. Images were processed in Fiji and Photoshop.

#### Contour Plots

Images were fit to the representative spinal cord sections using the landmark correspondence plugin in ImageJ. X–Y coordinates were acquired in ImageJ. Isoline plots were generated from X–Y scatterplots using Bivariant Kernel Density Estimation function (gkde2) with default setups in MATLAB. Nine isolines (from yellow to blue) were generated by default: yellow line, most dense region; blue line, least dense region.

### QUANTIFICATION AND STATISTICAL ANALYSIS

Statistical analysis was performed using Prism 7 software. Normality test was performed before sample comparison (Shapiro-Wilk normality test or D’Agostino & Pearson normality test). If samples were met normality criteria, samples were compared using two tailed Student’s t test; if not, non-parametric (Wilcoxon–Mann–Whitney) tests were used.

### DATA AND SOFTWARE AVAILABILITY

RNaseq data and analyses were deposited into the GEO repository under accession numbers GEO: GSE129948 and GEO: GSE130312.

## Supplementary Material

1

2

## Figures and Tables

**Table:** KEY RESOURCES TABLE

REAGENT or RESOURCE	SOURCE	IDENTIFIER
Antibodies		
guinea pig anti-Hoxc10	This paper	N/A
rabbit anti-Hoxc6	Aviva	Cat# ARP38484; RRID:AB_10866814
rabbit anti-Foxp2	Abcam	Cat# AB16046; RRID:AB_2107107
sheep anti-Lrrn1	R&D systems	Cat# AF4990; RRID:AB_2234807
rabbit anti-Chmp2b	Abcam	Cat# AB33174; RRID:AB_2079471
rabbit anti-Syt4	Synaptic systems	Cat# 105043; RRID:AB_887837
rabbit anti-Ebf3	Millipore	Cat# AB10525
rabbit anti-CTB	Sigma-Aldrich	Cat# C3062; RRID:AB_258833
goat anti-CTB	List Biological Lab	Cat# 703; RRID:AB_10013220
goat anti-ChAT	Millipore	Cat# AB144P; RRID:AB_2079751
guinea pig anti-VgluT1	Millipore	Cat# AB5905; RRID:AB_2301751
guinea pig anti-VgluT2	Millipore	Cat# AB2251; RRID:AB_2665454
goat anti-Scip	SantaCruz	Cat# SC11661; RRID:AB_2268536
rabbit anti-Shox2	Thomas Jessell	N/A
rabbit anti-GFP	Thermo Fisher	Cat# A-6455; RRID:AB_221570
rabbit anti-Hoxa5	Dasen et al., 2005	N/A
rabbit anti-Hoxa10	Dasen et al., 2005	N/A
rabbit anti-Hoxc4	Dasen et al., 2005	N/A
rabbit anti-Hoxc5	Dasen et al., 2005	N/A
mouse anti-Hoxa9	Dasen et al., 2005	N/A
guinea pig anti-Hoxc9	Jung et al., 2010	RRID: AB_2636809
guinea pig anti-Hoxc6	Liu et al., 2001	RRID: AB_528287
Alexa 647 anti-Rabbit antibody	Jackson ImmnoResearch	Cat# 711-605-152
Alexa 647 anti-Guinea Pig antibody	Jackson ImmnoResearch	Cat# 706-605-148
Alexa 647 anti-Mouse antibody	Jackson ImmnoResearch	Cat# 715-605-150
Cy3 anti-Guinea Pig antibody	Jackson ImmnoResearch	Cat# 706-165-148
Cy3 anti-Mouse antibody	Jackson ImmnoResearch	Cat# 715-165-150
Cy3 anti-Rabbit antibody	Jackson ImmnoResearch	Cat# 711-165-152
Alexa 488 anti-Rabbit antibody	Jackson ImmnoResearch	Cat# 711-545-152
Alexa 488 anti-Guinea pig antibody	Invitrogen	Cat# A11073
Alexa 488 anti-Mouse antibody	Invitrogen	Cat# A21202
anti-DIG-AP Fab fragments	Sigma-Aldrich	Cat# 11093274910
Bacterial and Virus Strains		
*AAV-SL1-synGFP*	Janelia Research Campus	N/A
Chemicals, Peptides, and Recombinant Proteins		
Horseradish peroxidase (HRP)	Sigma-Aldrich	Cat# 10814407001
20X SSC	Invitrogen	Cat# 15557–036
NBT	Sigma-Aldrich	Cat# 1383213
BCIP	Sigma-Aldrich	Cat# 1383221
Salmon Sperm DNA	Invitrogen	Cat# 15632–011
Yeast RNA	Invitrogen	Cat# AM7118
Paraformaldehyde	Sigma-Aldrich	Cat# 158127–500G
UltraPure Formamide	Invitrogen	Cat# 15515–026
Proteinase K	Sigma-Aldrich	Cat# 03115879001
Denhardt’s Solution (50X)	Invitrogen	Cat# 750018
Triethanolamine	Sigma-Aldrich	Cat# 33729–1L
Glycergel	Agilent	Cat# C0563
Vectashield	Vector Laboratories	Cat# H-1200
Fast Green	Sigma-Aldrich	Cat# F7258
Hoxc10 peptide: EFEAPFEQRASLNPRTEHC	Covance	This paper
Critical Commercial Assays		
DIG RNA Labeling Kit (SP6/T7)	Sigma-Aldrich	Cat# 11175025910
Ovation® RNA-Seq System V2	Nugen	Cat# 7102
Nugen Ovation Ultralow Library System	Nugen	Cat# 0303–05, Cat# 0330–31
PicoPure RNA Isolation Kit	Thermo Fisher	Cat# KIT0204
Deposited Data		
Bulk SCTN RNaseq data	GEO	GEO: GSE129948
scRNA-seq data SCTNs	GEO	GEO: GSE130312
Experimental Models: Organisms/Strains		
Mouse: *Hoxc9 -/-*	Jung et al., 2010	MGI:2447619; RRID:MGI:2447619
Mouse: *Hoxc8-/-;*	Catela et al., 2016	N/A
Mouse: *Nestin::Cre*	JAX	RRID:IMSR_JAX:003771
Mouse: *Hoxc9 flox/flox*	Baek et al., 2017	N/A
Oligonucleotides		
See Table S1	This paper	Table S1
Software and Algorithms		
STAR aligner	Dobin et al., 2013	http://code.google.com/p/rna-star/
edgeR v3.18.1	Robinson et al., 2010	http://bioconductor.org/packages/release/bioc/html/edgeR.html
Zen	Zeiss	https://www.zeiss.com/microscopy/us/products/microscope-software/zen.html
Prism v7.0c	Graphpad Software	https://www.graphpad.com/scientific-software/prism/

## Highlights

- Molecular and anatomical characterization of spinocerebellar tract neurons (SCTNs)
- Segment-specific Hox activity controls SCTN subtype diversification
- Mutation in Hoxc9 transforms the fate of thoracic Clarke’s column SCTNs
- Hox-dependent programs are essential for spinocerebellar circuit assembly

## References

[R1] Abelew TA, Miller MD, Cope TC, and Nichols TR (2000). Local loss of proprioception results in disruption of interjoint coordination during locomotion in the cat. J. Neurophysiol 84, 2709–2714. 11068014 10.1152/jn.2000.84.5.2709

[R2] Akay T, Tourtellotte WG, Arber S, and Jessell TM (2014). Degradation of mouse locomotor pattern in the absence of proprioceptive sensory feedback. Proc. Natl. Acad. Sci. USA 111, 16877–16882. 25389309 10.1073/pnas.1419045111 PMC4250167

[R3] Arber S, Ladle DR, Lin JH, Frank E, and Jessell TM (2000). ETS gene Er81 controls the formation of functional connections between group Ia sensory afferents and motor neurons. Cell 101, 485–498. 10850491 10.1016/s0092-8674(00)80859-4

[R4] Arsénio Nunes ML, and Sotelo C (1985). Development of the spinocerebellar system in the postnatal rat. J. Comp. Neurol 237, 291–306. 3840179 10.1002/cne.902370302

[R5] Baek M, Pivetta C, Liu JP, Arber S, and Dasen JS (2017). Columnar Intrinsic Cues Shape Premotor Input Specificity in Locomotor Circuits. Cell Rep 21, 867–877. 29069594 10.1016/j.celrep.2017.10.004 PMC5665584

[R6] Bangma GC, and ten Donkelaar H (1982). Afferent connections of the cerebellum in various types of reptiles. J. Comp. Neurol 207, 255–273. 7107986 10.1002/cne.902070306

[R7] Bermingham NA, Hassan BA, Wang VY, Fernandez M, Banfi S, Bellen HJ, Fritzsch B, and Zoghbi HY (2001). Proprioceptor pathway development is dependent on Math1. Neuron 30, 411–422. 11395003 10.1016/s0896-6273(01)00305-1

[R8] Betley JN, Wright CV, Kawaguchi Y, Erdélyi F, Szabó G, Jessell TM, and Kaltschmidt JA (2009). Stringent specificity in the construction of a GABAergic presynaptic inhibitory circuit. Cell 139, 161–174. 19804761 10.1016/j.cell.2009.08.027 PMC2812434

[R9] Bikoff JB, Gabitto MI, Rivard AF, Drobac E, Machado TA, Miri A, Brenner-Morton S, Famojure E, Diaz C, Alvarez FJ, (2016). Spinal Inhibitory Interneuron Diversity Delineates Variant Motor Microcircuits. Cell 165, 207–219. 26949184 10.1016/j.cell.2016.01.027 PMC4808435

[R10] Bosco G, and Poppele RE (2001). Proprioception from a spinocerebellar perspective. Physiol. Rev 81, 539–568. 11274339 10.1152/physrev.2001.81.2.539

[R11] Catela C, Shin MM, Lee DH, Liu JP, and Dasen JS (2016). Hox Proteins Coordinate Motor Neuron Differentiation and Connectivity Programs through Ret/Gfra Genes. Cell Rep 14, 1901–1915. 26904955 10.1016/j.celrep.2016.01.067 PMC4775310

[R12] Chen HH, Hippenmeyer S, Arber S, and Frank E (2003). Development of the monosynaptic stretch reflex circuit. Curr. Opin. Neurobiol 13, 96–102. 12593987 10.1016/s0959-4388(03)00006-0

[R13] Dasen JS (2009). Transcriptional networks in the early development of sensory-motor circuits. Curr. Top. Dev. Biol 87, 119–148. 19427518 10.1016/S0070-2153(09)01204-6

[R14] Dasen JS, Liu JP, and Jessell TM (2003). Motor neuron columnar fate imposed by sequential phases of Hox-c activity. Nature 425, 926–933. 14586461 10.1038/nature02051

[R15] Dasen JS, Tice BC, Brenner-Morton S, and Jessell TM (2005). A Hox regulatory network establishes motor neuron pool identity and target-muscle connectivity. Cell 123, 477–491. 16269338 10.1016/j.cell.2005.09.009

[R16] de Nooij JC, Doobar S, and Jessell TM (2013). Etv1 inactivation reveals proprioceptor subclasses that reflect the level of NT3 expression in muscle targets. Neuron 77, 1055–1068. 23522042 10.1016/j.neuron.2013.01.015 PMC3763960

[R17] Dietz V (2002). Proprioception and locomotor disorders. Nat. Rev. Neurosci 3, 781–790. 12360322 10.1038/nrn939

[R18] Dobin A, Davis CA, Schlesinger F, Drenkow J, Zaleski C, Jha S, Batut P, Chaisson M, and Gingeras TR (2013). STAR: ultrafast universal RNA-seq aligner. Bioinformatics 29, 15–21. 23104886 10.1093/bioinformatics/bts635 PMC3530905

[R19] Edgley SA, and Grant GM (1991). Inputs to spinocerebellar tract neurones located in stilling’s nucleus in the sacral segments of the rat spinal cord. J. Comp. Neurol 305, 130–138. 2033121 10.1002/cne.903050112

[R20] Francius C, Harris A, Rucchin V, Hendricks TJ, Stam FJ, Barber M, Kurek D, Grosveld FG, Pierani A, Goulding M, and Clotman F (2013). Identification of multiple subsets of ventral interneurons and differential distribution along the rostrocaudal axis of the developing spinal cord. PLoS ONE 8, e70325. 23967072 10.1371/journal.pone.0070325 PMC3744532

[R21] Ghez C, Gordon J, and Ghilardi MF (1995). Impairments of reaching movements in patients without proprioception. II. Effects of visual information on accuracy. J. Neurophysiol 73, 361–372. 7714578 10.1152/jn.1995.73.1.361

[R22] Gonzalez A, ten Donkelaar HJ, and de Boer-van Huizen R (1984). Cerebellar connections in Xenopus laevis. An HRP study. Anat. Embryol. (Berl.) 169, 167–176. 6742456 10.1007/BF00303146

[R23] Gordon J, Ghilardi MF, and Ghez C (1995). Impairments of reaching movements in patients without proprioception. I. Spatial errors. J. Neurophysiol 73, 347–360. 7714577 10.1152/jn.1995.73.1.347

[R24] Hantman AW, and Jessell TM (2010). Clarke’s column neurons as the focus of a corticospinal corollary circuit. Nat. Neurosci 13, 1233–1239. 20835249 10.1038/nn.2637 PMC2947611

[R25] Hayashi M, Hinckley CA, Driscoll SP, Moore NJ, Levine AJ, Hilde KL, Sharma K, and Pfaff SL (2018). Graded Arrays of Spinal and Supraspinal V2a Interneuron Subtypes Underlie Forelimb and Hindlimb Motor Control. Neuron 97, 869–884.e5. 29398364 10.1016/j.neuron.2018.01.023 PMC8601153

[R26] Hempel CM, Sugino K, and Nelson SB (2007). A manual method for the purification of fluorescently labeled neurons from the mammalian brain. Nat. Protoc 2, 2924–2929. 18007629 10.1038/nprot.2007.416

[R27] Inoue K, Ozaki S, Shiga T, Ito K, Masuda T, Okado N, Iseda T, Kawaguchi S, Ogawa M, Bae SC, (2002). Runx3 controls the axonal projection of proprioceptive dorsal root ganglion neurons. Nat. Neurosci 5, 946–954. 12352981 10.1038/nn925

[R28] Jung H, Lacombe J, Mazzoni EO, Liem KF Jr., Grinstein J, Mahony S, Mukhopadhyay D, Gifford DK, Young RA, Anderson KV, (2010). Global control of motor neuron topography mediated by the repressive actions of a single hox gene. Neuron 67, 781–796. 20826310 10.1016/j.neuron.2010.08.008 PMC2955411

[R29] Jung H, Baek M, D’Elia KP, Boisvert C, Currie PD, Tay BH, Venkatesh B, Brown SM, Heguy A, Schoppik D, (2018). The Ancient Origins of Neural Substrates for Land Walking. Cell 172, 667–682.e15. 29425489 10.1016/j.cell.2018.01.013 PMC5808577

[R30] Knox CK, Kubota S, and Poppele RE (1977). A determination of excitability changes in dorsal spinocerebellar tract neurons from spike-train analysis. J. Neurophysiol 40, 626–646. 874532 10.1152/jn.1977.40.3.626

[R31] Kuno M, Muñoz-Martinez EJ, and Randić M (1973). Sensory inputs to neurones in Clarke’s column from muscle, cutaneous and joint receptors. J. Physiol 228, 327–342. 4687101 10.1113/jphysiol.1973.sp010089 PMC1331300

[R32] Liu JP, Laufer E, and Jessell TM (2001). Assigning the positional identity of spinal motor neurons: rostrocaudal patterning of Hox-c expression by FGFs, Gdf11, and retinoids. Neuron 32, 997–1012. 11754833 10.1016/s0896-6273(01)00544-x

[R33] Mann MD (1973). Clarke’s column and the dorsal spinocerebellar tract: a review. Brain Behav. Evol 7, 34–83. 4349416 10.1159/000124397

[R34] Matsushita M, and Gao X (1997). Projections from the thoracic cord to the cerebellar nuclei in the rat, studied by anterograde axonal tracing. J. Comp. Neurol 386, 409–421. 9303426 10.1002/(sici)1096-9861(19970929)386:3<409::aid-cne6>3.0.co;2-5

[R35] Matsushita M, Hosoya Y, and Ikeda M (1979). Anatomical organization of the spinocerebellar system in the cat, as studied by retrograde transport of horseradish peroxidase. J. Comp. Neurol 184, 81–106. 84004 10.1002/cne.901840106

[R36] McCarthy DJ, Chen Y, and Smyth GK (2012). Differential expression analysis of multifactor RNA-Seq experiments with respect to biological variation. Nucleic Acids Res 40, 4288–4297. 22287627 10.1093/nar/gks042 PMC3378882

[R37] Mendell LM, and Henneman E (1968). Terminals of single Ia fibers: distribution within a pool of 300 homonymous motor neurons. Science 160, 96–98. 4296007 10.1126/science.160.3823.96

[R38] Mendelsohn AI, Simon CM, Abbott LF, Mentis GZ, and Jessell TM (2015). Activity Regulates the Incidence of Heteronymous Sensory-Motor Connections. Neuron 87, 111–123. 26094608 10.1016/j.neuron.2015.05.045 PMC4504246

[R39] Osborn CE, and Poppele RE (1988). The extent of polysynaptic responses in the dorsal spinocerebellar tract to stimulation of group I afferent fibers in gastrocnemius-soleus. J. Neurosci 8, 316–319. 3339414 10.1523/JNEUROSCI.08-01-00316.1988 PMC6569368

[R40] Pecho-Vrieseling E, Sigrist M, Yoshida Y, Jessell TM, and Arber S (2009). Specificity of sensory-motor connections encoded by Sema3e-Plxnd1 recognition. Nature 459, 842–846. 19421194 10.1038/nature08000 PMC2847258

[R41] Philippidou P, and Dasen JS (2013). Hox genes: choreographers in neural development, architects of circuit organization. Neuron 80, 12–34. 24094100 10.1016/j.neuron.2013.09.020 PMC3835187

[R42] Popova LB, Ragnarson B, Orlovsky GN, and Grant G (1995). Responses of neurons in the central cervical nucleus of the rat to proprioceptive and vestibular inputs. Arch. Ital. Biol 133, 31–45. 7748060

[R43] Robinson MD, McCarthy DJ, and Smyth GK (2010). edgeR: a Bioconductor package for differential expression analysis of digital gene expression data. Bioinformatics 26, 139–140. 19910308 10.1093/bioinformatics/btp616 PMC2796818

[R44] Rose MF, Ahmad KA, Thaller C, and Zoghbi HY (2009). Excitatory neurons of the proprioceptive, interoceptive, and arousal hindbrain networks share a developmental requirement for Math1. Proc. Natl. Acad. Sci. USA 106, 22462–22467. 20080794 10.1073/pnas.0911579106 PMC2799716

[R45] Sengul G, Fu Y, Yu Y, and Paxinos G (2015). Spinal cord projections to the cerebellum in the mouse. Brain Struct. Funct 220, 2997–3009. 25009313 10.1007/s00429-014-0840-7

[R46] Shrestha SS, Bannatyne BA, Jankowska E, Hammar I, Nilsson E, and Maxwell DJ (2012). Excitatory inputs to four types of spinocerebellar tract neurons in the cat and the rat thoraco-lumbar spinal cord. J. Physiol 590, 1737–1755. 22371473 10.1113/jphysiol.2011.226852 PMC3413493

[R47] Sürmeli G, Akay T, Ippolito GC, Tucker PW, and Jessell TM (2011). Patterns of spinal sensory-motor connectivity prescribed by a dorsoventral positional template. Cell 147, 653–665. 22036571 10.1016/j.cell.2011.10.012 PMC3238499

[R48] Sweeney LB, Bikoff JB, Gabitto MI, Brenner-Morton S, Baek M, Yang JH, Tabak EG, Dasen JS, Kintner CR, and Jessell TM (2018). Origin and Segmental Diversity of Spinal Inhibitory Interneurons. Neuron 97, 341–355.e3. 29307712 10.1016/j.neuron.2017.12.029 PMC5880537

[R49] Tasic B, Yao Z, Graybuck LT, Smith KA, Nguyen TN, Bertagnolli D, Goldy J, Garren E, Economo MN, Viswanathan S, (2018). Shared and distinct transcriptomic cell types across neocortical areas. Nature 563, 72–78. 30382198 10.1038/s41586-018-0654-5 PMC6456269

[R50] Tourtellotte WG, and Milbrandt J (1998). Sensory ataxia and muscle spindle agenesis in mice lacking the transcription factor Egr3. Nat. Genet 20, 87–91. 9731539 10.1038/1757

[R51] Tuthill JC, and Azim E (2018). Proprioception. Curr. Biol 28, R194–R203. 29510103 10.1016/j.cub.2018.01.064

[R52] Vrieseling E, and Arber S (2006). Target-induced transcriptional control of dendritic patterning and connectivity in motor neurons by the ETS gene Pea3. Cell 127, 1439–1452. 17190606 10.1016/j.cell.2006.10.042

[R53] Windhorst U (2007). Muscle proprioceptive feedback and spinal networks. Brain Res. Bull 73, 155–202. 17562384 10.1016/j.brainresbull.2007.03.010

[R54] Yuengert R, Hori K, Kibodeaux EE, McClellan JX, Morales JE, Huang TP, Neul JL, and Lai HC (2015). Origin of a Non-Clarke’s Column Division of the Dorsal Spinocerebellar Tract and the Role of Caudal Proprioceptive Neurons in Motor Function. Cell Rep 13, 1258–1271. 26527010 10.1016/j.celrep.2015.09.064 PMC4644487

