# Supplementary material for: Molecular Logic of Spinocerebellar Tract Neuron Diversity and Connectivity
Source: Cell Rep. Author manuscript; Available in PMC 2019 Jun 7. (PMC6555431; doi:10.1016/j.celrep.2019.04.113)
Supplement: 1 [file NIHMS1530455-supplement-1.pdf]

Cell Reports, Volume *27*

Supplemental Information

Molecular Logic of Spinocerebellar Tract Neuron Diversity and Connectivity

Myungin Baek, Vilas Menon, Thomas M. Jessell, Adam W. Hantman, and Jeremy S. Dasen

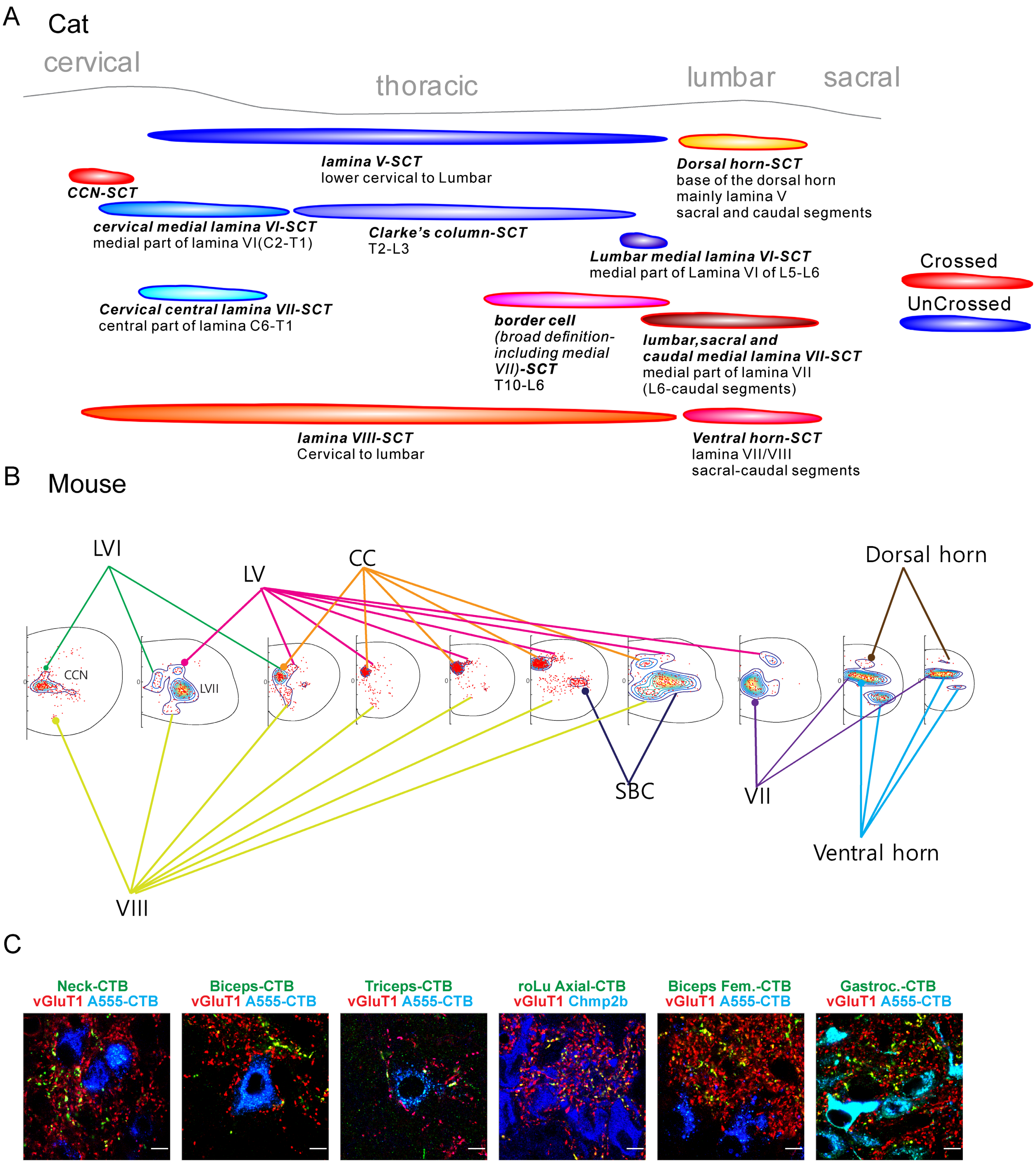

Figure S1. Diversity and Anatomical Location of SCTNs in Cat and Mouse. Related to Figure 1.
(A) Organization and diversity of SCTN subtypes described in cat. Diagram is based on data in Matsushita et al., 1979.
(B) Distribution and identity of SCTN subtypes in early postnatal mice.
(C) High magnification images of pSN inputs to SCTNs. Neurons were traced by CTB injection into indicated target muscle. Shown are the magnified images of regions demarked in Figure 1D. VGluT1 labels pSN terminals; A555-CTB labels traced SCTNs, Chmp2b marks Clarke's column neurons. Scale bars = 10 µm.

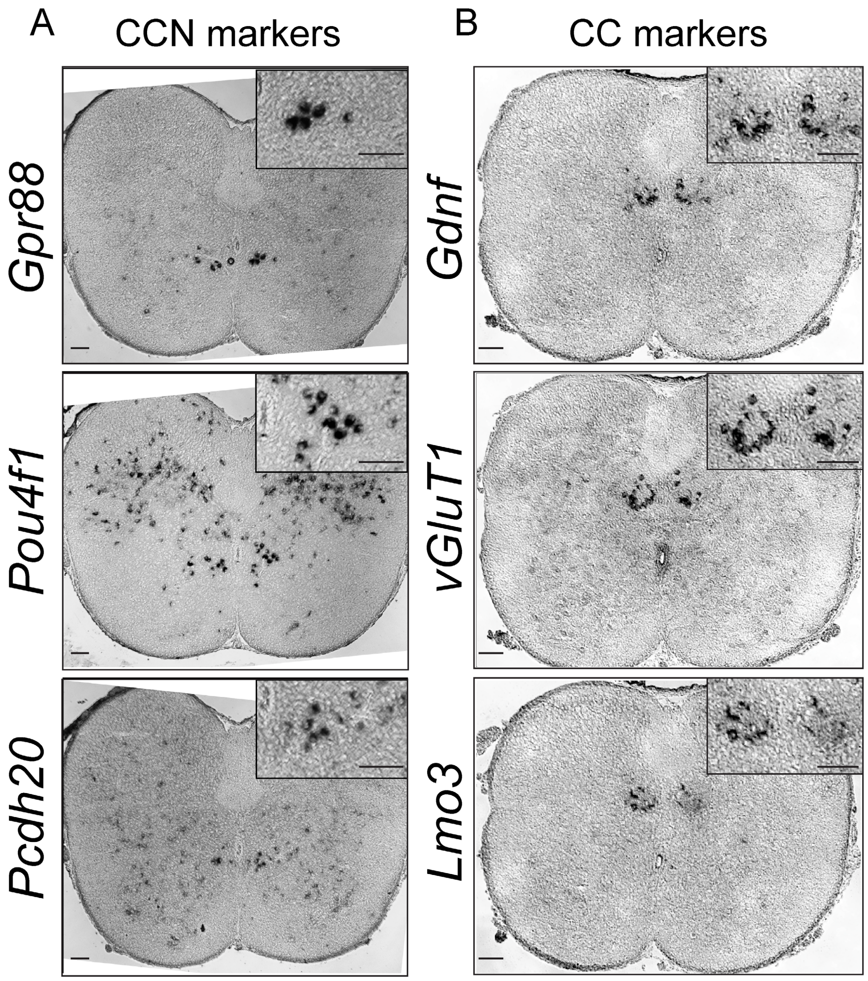

Figure S2. Expression of SCTN-Restricted Genes. Related to Figure 2.
(A) Expression of indicated CCN-restricted genes at rostral cervical levels. Scale bars = 100 µm.
(B) Expression of indicated CC-restricted genes at thoracic levels. Scale bars = 100 µm.

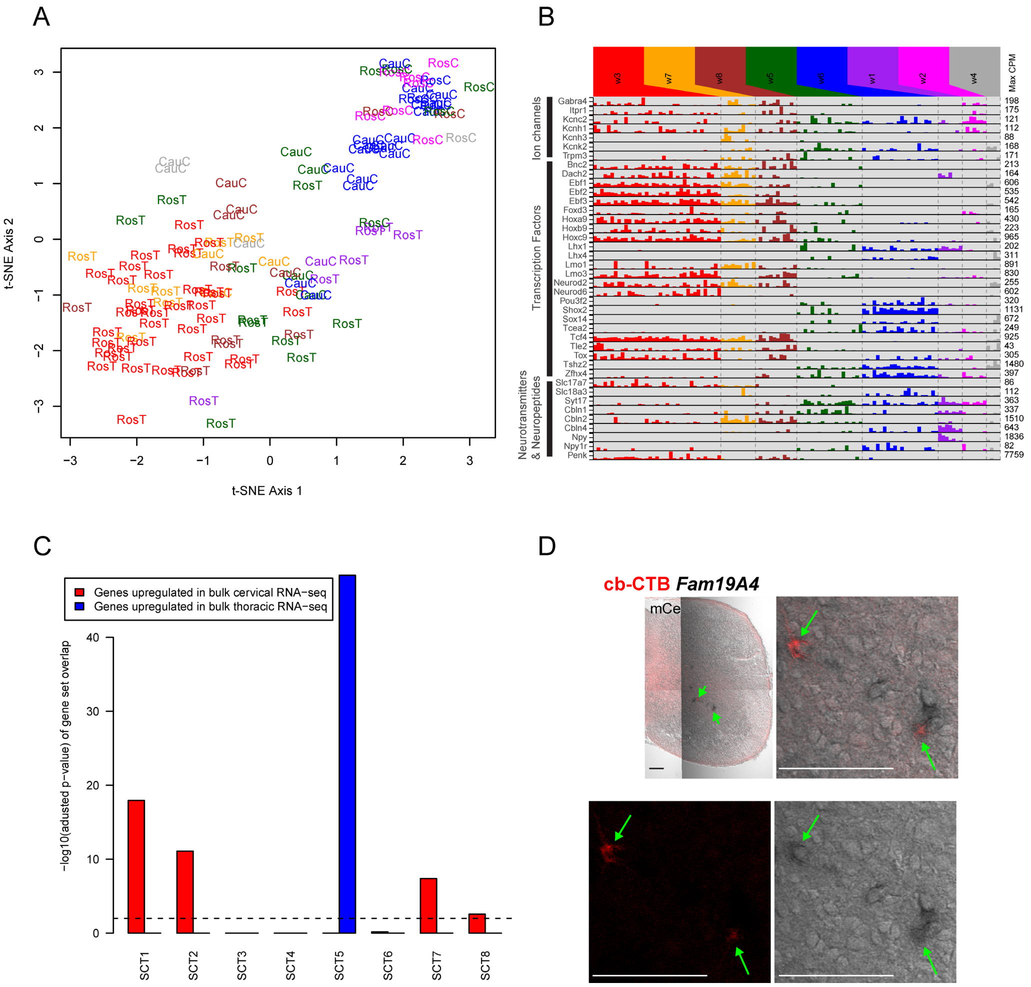

Figure S3. Analysis of SCTN scRNAseq Data. Related to Figure 3.
(A) tSNE visualization of scRNAseq data from control cells, using only *Hox* genes for dimensionality reduction, showing segmental origin of single cells. Cells are colored by their original clusters (using all genes, as reflected in Figure 3). RosT, rostral thoracic; RosC, rostral cervical; CauC, caudal cervical.
(B) Barplot showing expression (TPM) values of selected ion channels, transcription factors, and neurotransmitters within clusters.
(C) Comparison of bulk and scRNAseq data. The barplot shows the -log10 Bonferroni-adjusted p-value of the gene set overlap among cluster-specific genes from single-cell RNA-seq and differentially expressed genes from bulk RNA-seq of cervical and thoracic cells. Bulk RNA-seq genes were selected based on FDR<0.05 & fold-change>2. Cluster-specific gene sets from the single-cell RNA-seq were obtained for each cluster as follows: 1) for a given cluster, identify all genes upregulated in that cluster (FDR<0.05, fold-change>2) versus any other cluster using pairwise cluster comparisons, 2) select genes only if they are uniquely upregulated in that cluster i.e. no other cluster has significant upregulation of that gene with respect to any other cluster. P-values for each gene set overlap (8 cluster gene sets x 2 bulk gene = 16 overlaps in total) were calculated using the hypergeometric distribution, with the background gene set comprising all genes with any detection (>0) in either the single-cell or the bulk RNA-seq data. Gene set overlap p-values were adjusted post-hoc using the Bonferroni correction. As shown in the barplot, SCT1, SCT2, SCT7, and SCT8-specific gene sets show significant (p<0.01, dashed line) overlaps with genes upregulated in the bulk cervical RNA-seq data, whereas SCT5 expresses genes preferentially upregulated in the bulk thoracic RNA-seq data.
(D) Validation of *Fam19A4* as cLVII SCTN marker. *Fam19A4* in situ hybridization (shown in black) was performed on Cb-CTB traced tissue sections (shown in red). Top left panel is a composite of tiled images. Scale bars = 100 µm.

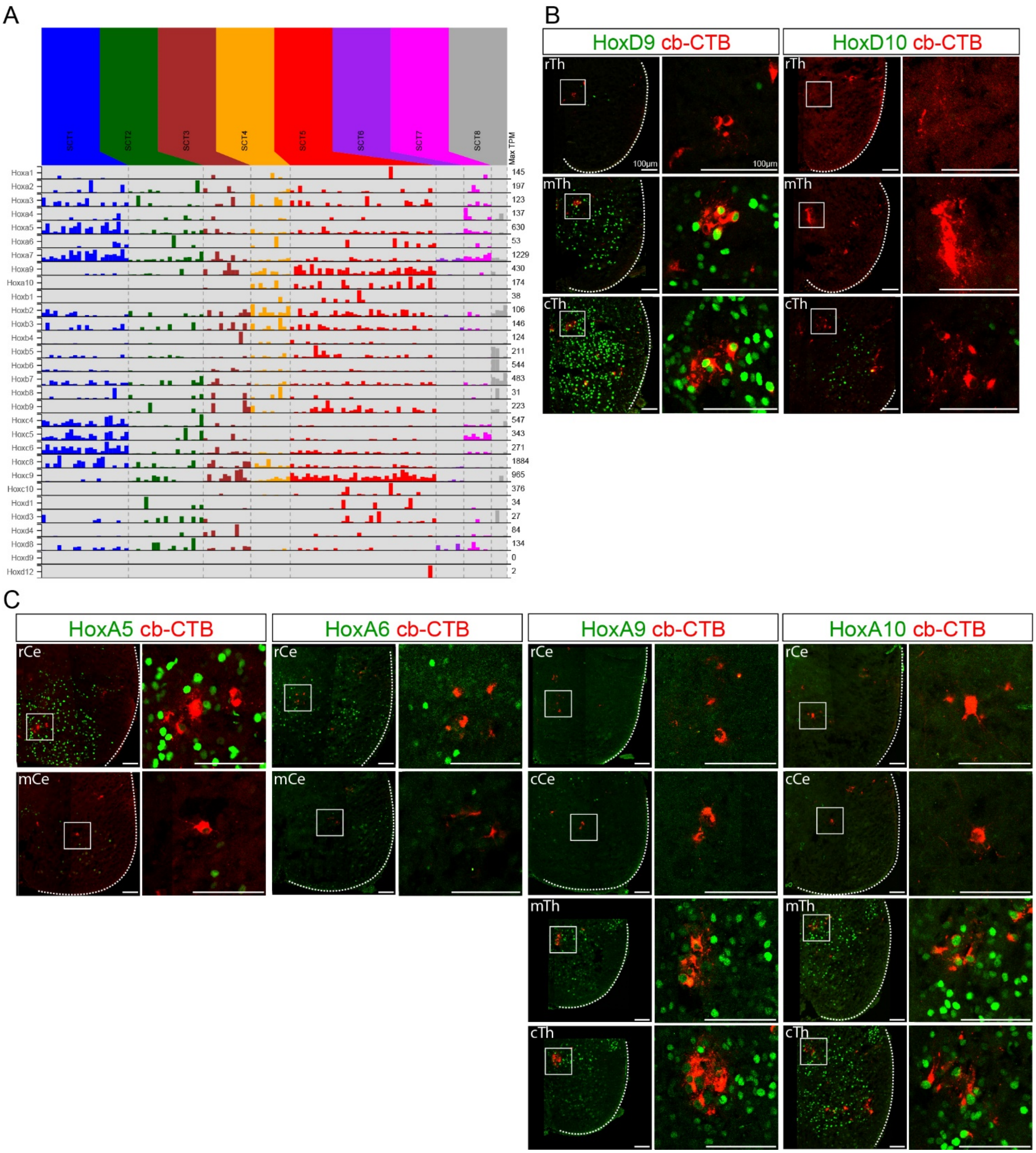

Figure S4. *Hox* Expression in SCTNs. Related to Figure 4.
(A) Barplot showing the expression (TPM) of *Hox* genes from each of the four clusters in the control scRNAseq dataset, arranged by cluster identity (SCT1 through SCT8).
(B) Expression of Hoxd9 and Hoxd10 in rTh, mTh, and cTh segments. SCTNs were labeled through CTB injection into the cerebellum. Scale bars = 100 µm.
(C) Expression of indicated HoxA proteins in rCe, mCe, mTh, and cTh segments. SCTNs were labeled through CTB injection into the cerebellum. Scale bars = 100 µm.

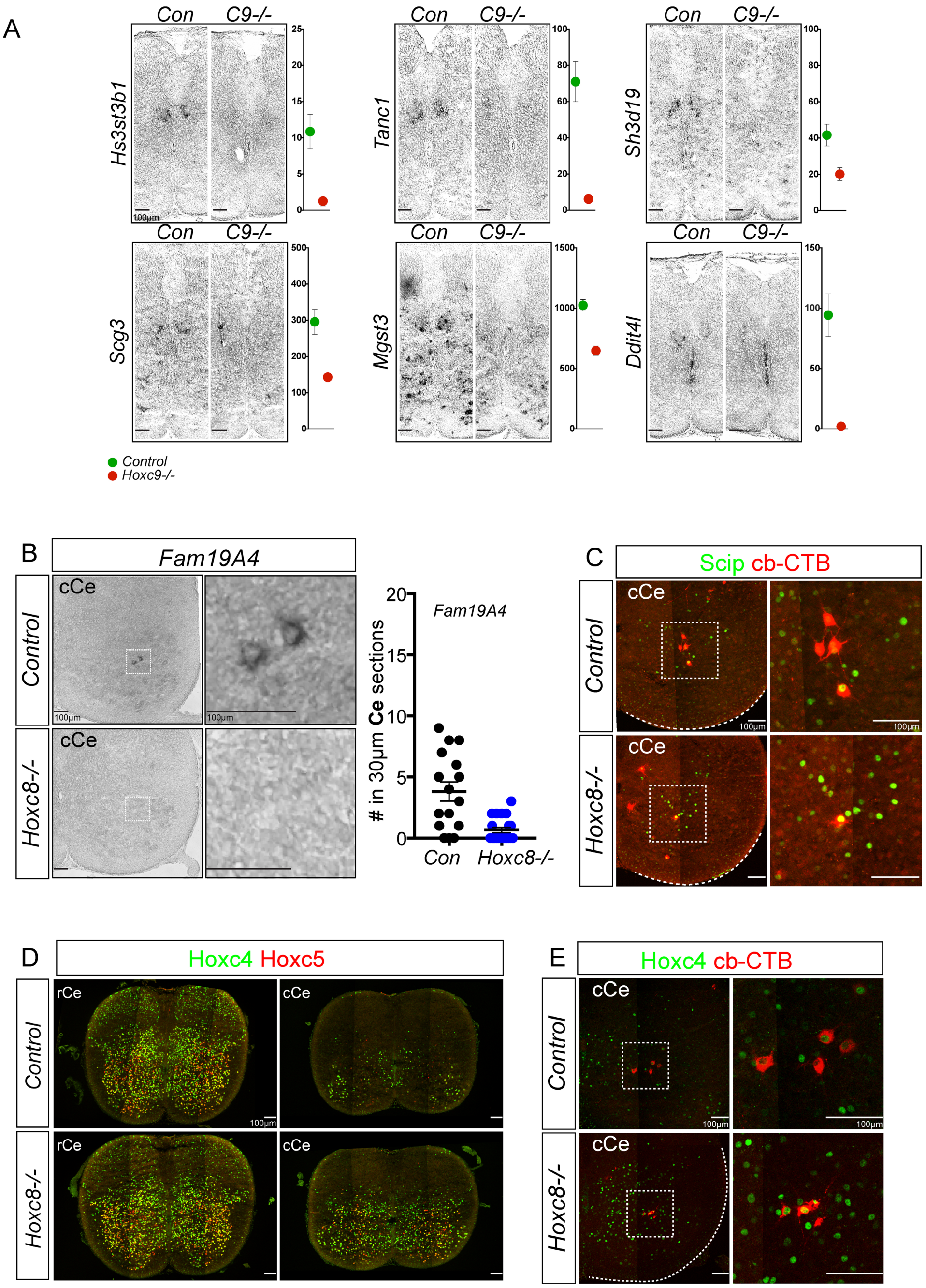

Figure S5. Analysis of SCNT Specification in *Hoxc9* and *Hoxc8* Mutants. Related to Figure 5.

(A) Analysis of CC neuron marker expression in thoracic segments of *Hoxc9* mutants. Expression levels from scRNAseq data in control and *Hoxc9-/-* thoracic SCTNs is shown on the right. Scale bars = 100 µm.

(B) *Fam19A4* expression in *Hoxc8* mutants. Two tailed Student's t test, p=0.0001 (***); Con, n=3 (P0, 2; P6, 1; 16 sections); *Hoxc8-/-*, n=4 (P0, 1; P6, 3; 21 sections). Scale bars = 100 µm.

(C) Expression of Scip in SCTNs of *Hoxc8* mutants. Panels are composites of tiled images. Scale bars = 100 µm.

(D) Hoxc4 and Hoxc5 protein expression at P0 in *Hoxc8* mutants. Both Hoxc4 and Hoxc5 are derepressed in cCe segments of *Hoxc8* mutants. Scale bars = 100 µm.

(E) Caudal cervical SCTNs ectopically express Hoxc4 in *Hoxc8* mutants. Scale bars = 100 µm.

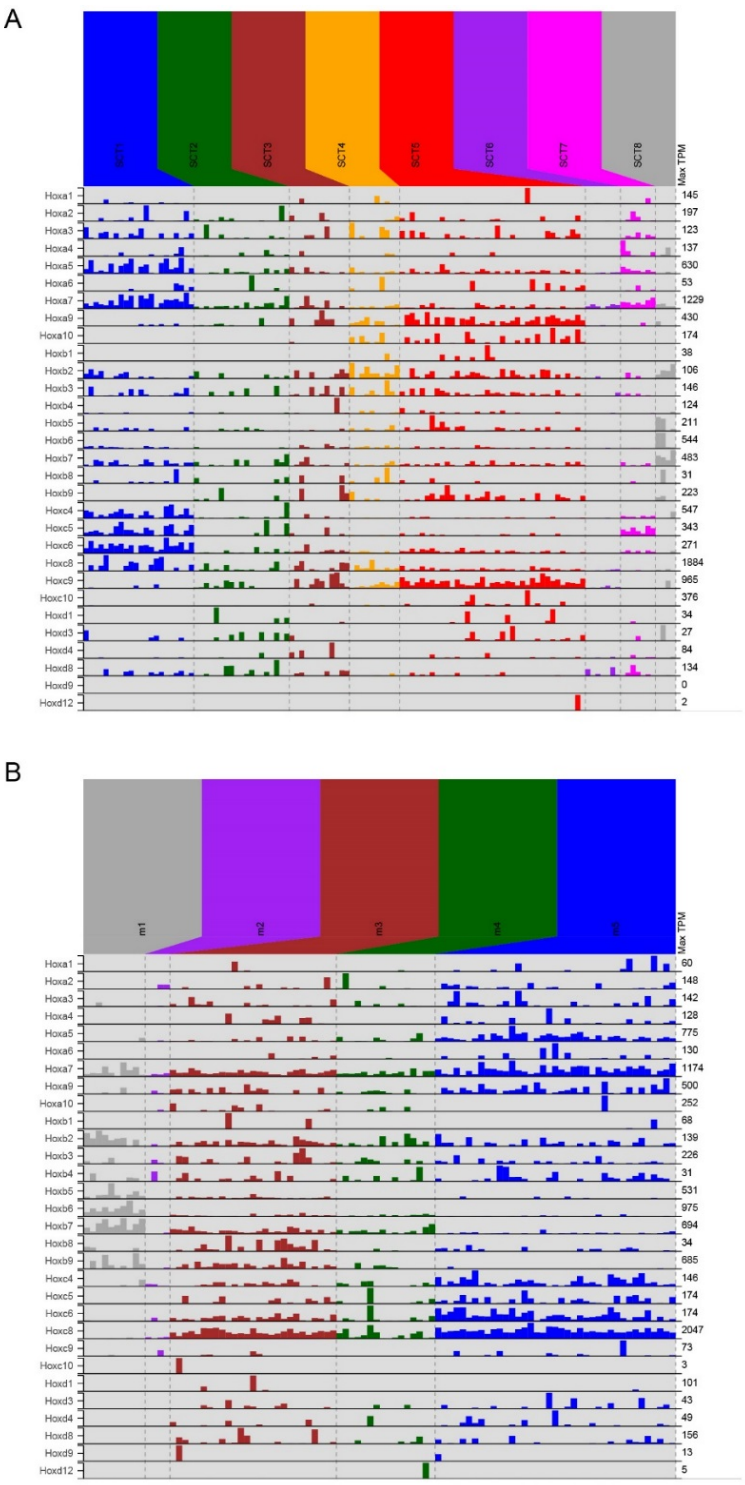

Figure S6. Expression of *Hox* Genes in Control and *Hoxc9* Mutant SCTNs. Related to Figure 6.
(A) Barplot showing the expression (TPM) of *Hox* genes in the control scRNA-seq data, arranged by cluster identity (SCT1 through SCT8). Plot is identical to Figure S4a, and is shown here for direct comparison to *Hoxc9* mutants.
(B) Same as panel A, but for *Hoxc9* mutant cells, arranged by mutant cluster identity. For both panels, *Hox* genes with no detected expression (in control or *Hoxc9* mutant cells) are not shown.

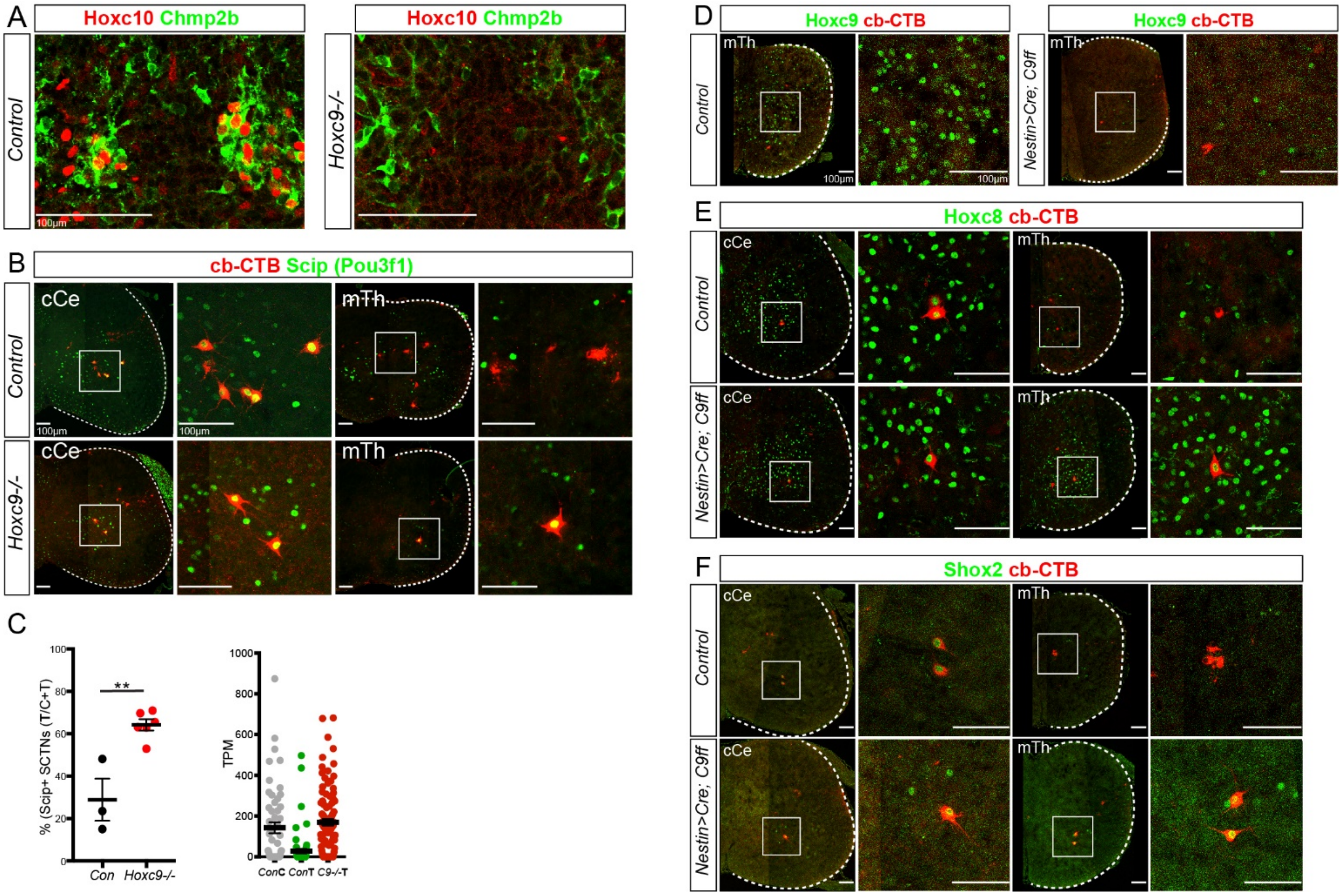

Figure S7. SCTN Differentiation in *Hoxc9-/-* and *Nestin::Cre; Hoxc9 flox/flox* Mice. Related to Figure 7.
(A) Loss of Hoxc10 protein expression in thoracic segments of *Hoxc9* mutants. Chmp2b marks CC neurons.
(B) Ectopic expression of Scip (Pou3f1) in thoracic SCTNs of *Hoxc9* mutants.
(C) Quantification of Scip^{+} SCTN number and TPM values in single cells from control cervical (ConC), control thoracic (ConT) and *Hoxc9* mutant thoracic (C9-/-T) regions. For the Scip+ cell quantification cells were counted in regions belonging to the LVII group according to contour plot in Figure 1. Two tailed Student's t test, p=0.0023 (**); Con, n=3, 81 cells (Ce, 57; Th, 24); *Hoxc9-/-*, n=6, 156 cells (Ce, 54; Th,102).
(D) Loss of Hoxc9 protein expression in thoracic segments of *Nestin::Cre; Hoxc9 flox/flox* mice.
(E) In *Hoxc9* conditional mutants, SCTNs at rostral thoracic levels acquire Hoxc8 expression.
(F) Rostral thoracic SCTNs ectopically express Shox2 in *Nestin::Cre; Hoxc9 flox/flox* mice. Images are composites of tiled images. Scale bars in panels A, B, D, E, F= 100 µm.

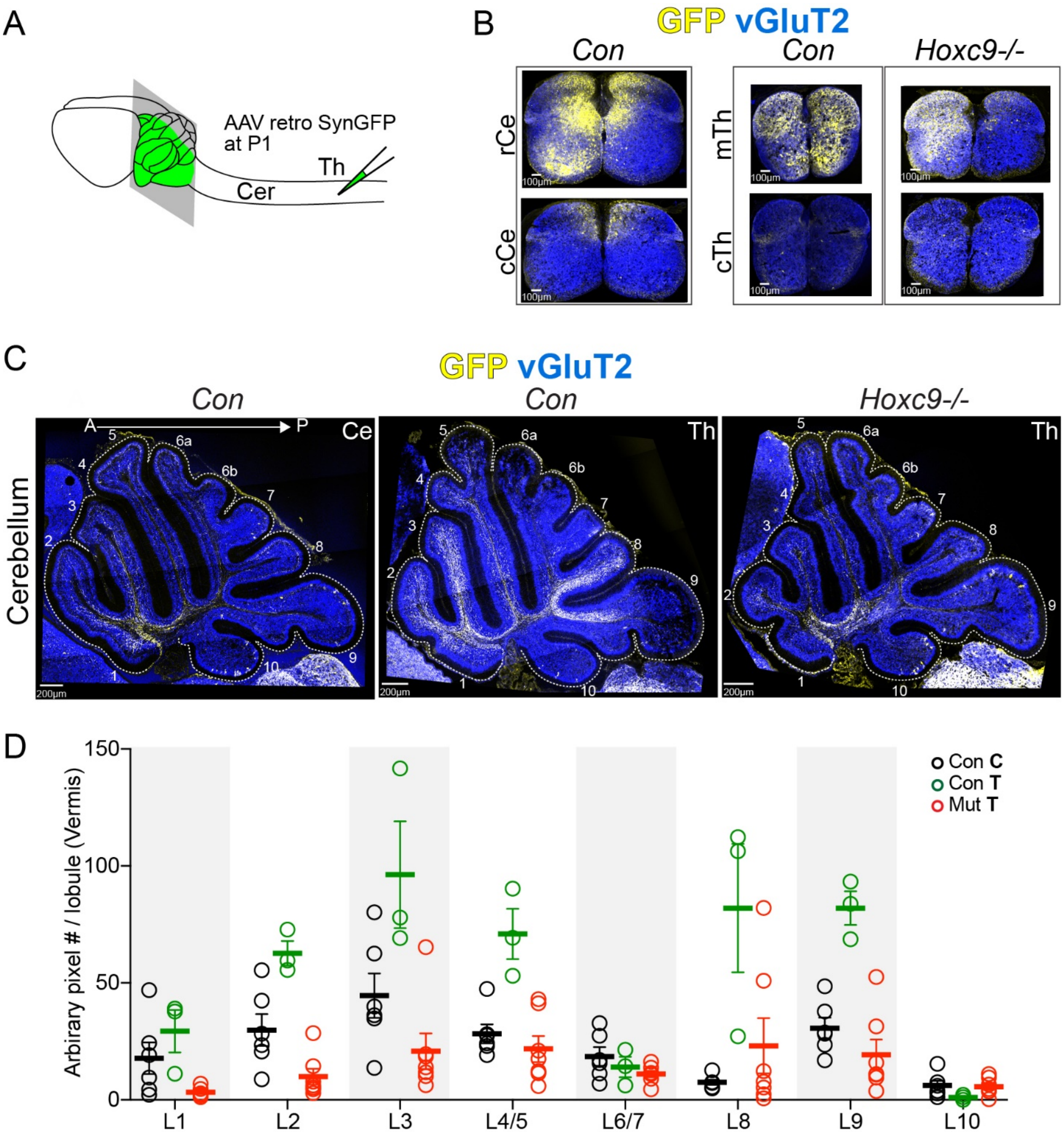

Figure S8. Analysis of Spinal Projections to Cerebellum in *Hoxc9* Mutants. Related to Figure 7.
(A) Strategy for spinal cord injection of *AAV-retro::SynGFP*. Virus was injected at P1 and GFP signals were examined at P6.
(B) Analysis of GFP expression within indicated spinal cord sections of control and *Hoxc9* mutant mice. Scale bars = 100 µm.
(C) Analysis of GFP expression in the cerebellum. Cerebellar lobule numbers are indicated. Scale bars = 500 µm.
(D) Quantification of projections within the vermis. In *Hoxc9* mutants there is a relatively marked reduction in the innervation of lobule 8. GFP pixel number were counted. Con Ce, n=5 mice; Con Th, n=3 mice; *Hoxc9* -/- (Mut) Th, n=5 mice.

Table S1. Oligonucleotide Sequences Used to Amplify *In Situ* Probes

Sequences in blue: T7 RNA polymerase promoter sequence introduced at the 5'-end of antisense primer.

Genes		Primer sequences (5'->3')
Pou4f1	*Forward*	TACCGGGGATAAATGTTGAGTC
	T7-Reverse	TAATACGACTCACTATAGGG ATGGACAGGAGGATCAGTCAGT
Gpr88	*Forward*	AACCCGCTGCTCTACACG
	T7-Reverse	TAATACGACTCACTATAGGG GCTCCCCCTGTTTTTGCT
Pcdh20	*Forward*	ATCAGTGTAACAGATGCCGATG
	T7-Reverse	TAATACGACTCACTATAGGG CTTCCCTGTGATGCTTTTTACC
Ndnf	*Forward*	GCGATGCACCTTTGGAGT
	T7-Reverse	TAATACGACTCACTATAGGG GACAGAAGCAGCCTCCCA
vGluT1	*Forward*	CAGAGCCGGAGGAGATGA
	T7-Reverse	TAATACGACTCACTATAGGG TTCCCTCAGAAACGCTGG
Unc5c	*Forward*	GAGGCCTATTTAATTGTGGCTG
	T7-Reverse	TAATACGACTCACTATAGGG TCAACTGGCTCCTCTTTCTTTC
Enc1	*Forward*	CTTTGGTCTCGGCTGCTG
	T7-Reverse	TAATACGACTCACTATAGGG AACTATGGAGCGGGGGAC
Rgs4	*Forward*	ATGGCCTTCCCTCCTTTG
	T7-Reverse	TAATACGACTCACTATAGGG GGGAGCTCTGGGGACATT
AF529169	*Forward*	ATGCCTTACCACAACCAATACC
	T7-Reverse	TAATACGACTCACTATAGGG TTCGTGCTTCTCTAGGTCAACA
Sipa1l2	*Forward*	CATGGACAAGAACTAGAAGGGG
	T7-Reverse	TAATACGACTCACTATAGGG AACAGCGTTCGTGCTAGTGTTA
Sh3d19	*Forward*	CATGACAGGGAGGGCATC
	T7-Reverse	TAATACGACTCACTATAGGG GACAAATGGGGAACGCTG
Scg3	*Forward*	TCTCCAGAGGAAGGCGTG
	T7-Reverse	TAATACGACTCACTATAGGG ATTTCCGTGCCCAACAGA
Mgst3	*Forward*	GGTGAGCCAGAGCCAAGA
	T7-Reverse	TAATACGACTCACTATAGGG ATCAGGGTGAGTCGCTGG
Rspo2	*Forward*	ACAGGAGGCACAAGGCTG
	T7-Reverse	TAATACGACTCACTATAGGG TTGGGCGAAGCCATTCTA
Id4	*Forward*	GGCCAGAGCAGAAATTAAGAGA
	T7-Reverse	TAATACGACTCACTATAGGG GAAACTGGATACTGGGCAAAAC

Sulf1	*Forward*<br>*T7-Reverse*	TGGGAAGGTTAGTCAGTCCAAT<br>TAATACGACTCACTATAGGG TCTGTCAGAGTGGACAAGAGGA
Lrrn1	*Forward*<br>*T7-Reverse*	TTCCACTCCCAACCTGGA<br>TAATACGACTCACTATAGGG CCAATGGGAGTGACCCAG
Syt4	*Forward*<br>*T7-Reverse*	CAGTGCTTTTGGCCTCGT<br>TAATACGACTCACTATAGGG CGTCCATGGCTGGTAAGC
Lmo3	*Forward*<br>*T7-Reverse*	CTGAATAGGGTAGCGGTAGGTG<br>TAATACGACTCACTATAGGG GAAGTTGTTCACATGCTACCCA
Sfrp2	*Forward*<br>*T7-Reverse*	AGCAACTGCAAGCCCATC<br>TAATACGACTCACTATAGGG ATGGAGAGAAGCCACCCC
Tanc1	*Forward*<br>*T7-Reverse*	TACAACCCAGGTGCAGGG<br>TAATACGACTCACTATAGGG TCCCTCCCCATCTTTTCC
Mllt3	*Forward*<br>*T7-Reverse*	ACGTTGCCACCGTTTGAT<br>TAATACGACTCACTATAGGG TGGGGATTTCCTTGCAGA
NeuroD6	*Forward*<br>*T7-Reverse*	GTCCTTCGAGGAAAGAGCATTA<br>TAATACGACTCACTATAGGGCAAGGTTTCTTCTTGCTTCAGG
Pvalb	*Forward*<br>*T7-Reverse*	TCTGCTCATCCAAGTTGCAG<br>TAATACGACTCACTATAGGGTCCTGAAGGACTCAACCCC
Ddit4l	*Forward*<br>*T7-Reverse*	GGCTTGCTGCAGGACTGT<br>TAATACGACTCACTATAGGGTGTCGTTCCAATCAGGGAG
Chmp2b	*Forward*<br>*T7-Reverse*	GATTGGTAATAAGGAAGCGTGC<br>TAATACGACTCACTATAGGGTACCTAAGAGCCAAACTGCTCC
Hs3st3b1	*Forward*<br>*T7-Reverse*	CAGAGAAGTCCCCAGCTTTGC<br>TAATACGACTCACTATAGGGGGTGTCCAGCTTGGAAAGAG
Fam19A4	*Forward*<br>*T7-Reverse*	AGCCTGGAGCCACCCTGGAC<br>TAATACGACTCACTATAGGGGATCAGGTGGTGACCTGCATG
